# The Hydrogen-Coupled Oligopeptide Membrane Cotransporter Pept2 is SUMOylated in Kidney Distal Convoluted Tubule Cells

**DOI:** 10.3389/fmolb.2021.790606

**Published:** 2021-11-22

**Authors:** Takwa S. Aroankins, Sathish K. Murali, Robert A. Fenton, Qi Wu

**Affiliations:** ^1^ Department of Biomedicine, Aarhus University, Aarhus, Denmark; ^2^ Department of Anesthesiology and Intensive Care, Sahlgrenska University Hospital, Sahlgrenska Academy at University of Gothenburg, Gothenburg, Sweden

**Keywords:** SUMOylation, kidney, distal convoluted tubule, proteomics, Pept2, aldosterone, membrane trafficking

## Abstract

Protein post-translational modification by the Small Ubiquitin-like MOdifier (SUMO) on lysine residues is a reversible process highly important for transcription and protein stability. In the kidney, SUMOylation appears to be important for the cellular response to aldosterone. Therefore, in this study, we generated a SUMOylation profile of the aldosterone-sensitive kidney distal convoluted tubule (DCT) as a basis for understanding SUMOylation events in this cell type. Using mass spectrometry-based proteomics, 1037 SUMO1 and 552 SUMO2 sites, corresponding to 546 SUMO1 and 356 SUMO2 proteins, were identified from a modified mouse kidney DCT cell line (mpkDCT). SUMOylation of the renal hydrogen-coupled oligopeptide and drug co-transporter (Pept2) at one site (K139) was found to be highly regulated by aldosterone. Using immunolabelling of mouse kidney sections Pept2 was localized to DCT cells *in vivo*. Aldosterone stimulation of mpkDCT cell lines expressing wild-type Pept2 or mutant K139R-Pept2, post-transcriptionally increased Pept2 expression up to four-fold. Aldosterone decreased wild-type Pept2 abundance in the apical membrane domain of mpkDCT cells, but this response was absent in K139R-Pept2 expressing cells. In summary, we have generated a SUMOylation landscape of the mouse DCT and determined that SUMOylation plays an important role in the physiological regulation of Pept2 trafficking by aldosterone.

## Introduction

Post-translational modification (PTM) of proteins by the Small Ubiquitin-like MOdifier (SUMO) on lysine residues is a reversible process highly important for genome activity, protein stability and transcription (Hay, 2005). There are five known SUMO family members expressed in mammals, SUMO1-5, with SUMO1-3 being the most abundant (Bohren et al., 2004; Liang et al., 2016; Ma et al., 2019) and high homology between SUMO2 and SUMO3 (Geiss-Friedlander and Melchior, 2007). The covalent conjugation of SUMO onto a target lysine (K) occurs via an enzyme cascade, including a dimeric E1 activating enzyme, a single E2 conjugating enzyme (Ubc9) and several E3 SUMO protein ligases. SUMO specific proteases (sentrin specific proteases, SENP) mature SUMOs and rapidly de-SUMOylate target proteins in response to cellular stress (Matic et al., 2010; Becker et al., 2013; Flotho and Melchior, 2013; Hendriks and Vertegaal, 2016). Classically SUMOylation sites have a consensus sequence of ψKxE, where ψ represents a bulky hydrophobic amino acid, but further studies have indicated that at least four types of sequence motifs exist (Yang and Chiang, 2013).

Proteomic studies of SUMOylation have generated large volumes of data that allows better understanding of the biological roles of SUMOylation (Hendriks and Vertegaal, 2016; Hendriks et al., 2017). The SUMO field continues to expand at a rapid pace, with over 180 studies in the past year alone demonstrating the role of SUMOylation in human pathophysiological conditions. For example, targeting SUMOylation may have clinical implications for cancer treatment (Ma et al., 2021), including prostate cancer (Wang and Yu, 2021), colorectal cancer (Peng et al., 2021), hematological malignancies (Wang et al., 2021a), bone chondrosarcoma (Kroonen et al., 2021) and breast cancer (Hu et al., 2021). SUMOylation is also important for endocrinal thyroid hormone production (Ke et al., 2021), has a cardiac protective role in heart failure and diabetic cardiomyopathy (Liu et al., 2021; Wang et al., 2021b) and plays a role in immunological responses and viral therapy (Imbert and Langford, 2021; Sajeev et al., 2021).

In the kidney, SUMOylation appears to reduce progression of renal cell carcinoma (Dong et al., 2016) and play a cytoprotective role in acute kidney injury (Guo et al., 2015). Increased SUMOylation has also been linked to progression of diabetic nephropathy, renal fibrosis and podocyte injury processes, all of which involve inflammatory response and cellular oxidative stresses (Li et al., 2019). SUMOylation also appears to play a role in modulating the renal response to the mineralocorticoid aldosterone, with both the mineralocorticoid receptor (MR) and the glucocorticoid metabolizing enzyme 11beta-hydroxysteroid dehydrogenase type 2 (11B-HSD2) being SUMOylated (Briet and Schiffrin, 2010). The ability of the MR to act as a transcription factor after binding to aldosterone (Yokota et al., 2007; Jimenez-Canino et al., 2017) fits well with the role of SUMOylation for modulating gene transcription. The kidney distal convoluted tubule (DCT) is one tubule region where aldosterone exerts its effects (Hoorn et al., 2020). To better understand the role of SUMOylation in this segment under physiological and pathophysiological conditions, here we used an established LC-MS/MS method (Tammsalu et al., 2015) to generate a landscape of SUMOylated proteins in an established model of the mouse distal convoluted tubule - mpkDCT cells (Peng et al., 1999; Cheng et al., 2015). Subsequently, we determined that SUMOylation of K139 in the hydrogen-coupled oligopeptide membrane cotransporter Pept2 (encoded by *slc15a2* gene) (Daniel and Rubio-Aliaga, 2003) is increased by aldosterone and plays a role in apical plasma membrane localization of the transporter.

## Materials and Methods

### Cell Culture and Transfection of Cell Lines

Mouse kidney distal convoluted tubule cells (mpkDCT) were cultured as described before (Wu et al., 2018). Cells were stably transfected using standard protocols for Lipofectamine 2000 (Invitrogen), with pEFIRESpuro vectors containing N-terminal hexa-histidine tagged SUMO1 (6xHis-SUMO1^T95K^) and SUMO2 (6xHis-SUMO2^T90K^) (Tammsalu et al., 2015). Stably expressing cells were selected using 2 µg/ml puromycin (Sigma-Aldrich) and named mpkDCT-SUMO1^T95K^ or mpkDCT-SUMO2^T90K^ cells. mpkDCT cells were also transfected with wild-type or K139R-mutant FLAG-tagged Pept2 (Genscript, DNA sequences in Supplementary Information) using Lipofectamine 2000 (Invitrogen). Stably expressing cells, named mpkDCT-Pept2 and mpkDCT-Pept2^K139R^, were selected using 350 µg/µl gentamicin.

### Cell Experiments

**FIGURE 1:**
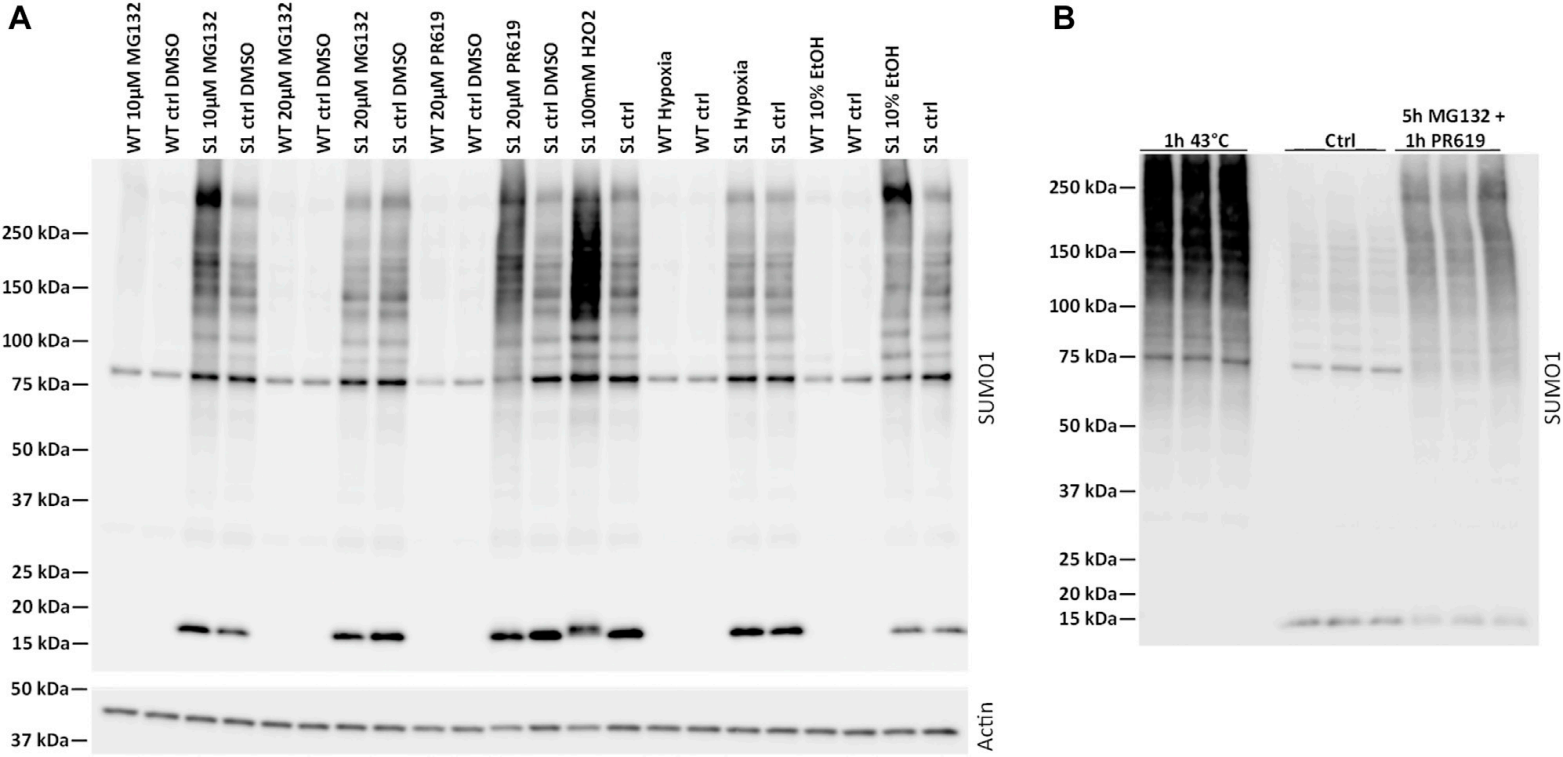
Characterization of SUMOylation in mpkDCT cells. **(A)** Wild-type (WT) and 6xHis-SUMO1^T95K^ mutated mpkDCT cells (mpkDCT-SUMO1^T95K^) were treated with various chemicals or pathophysiological conditions known to increase protein SUMOylation. After inhibition of deSUMOylation enzymes with PR619 or MG132, or inducing cell stress with hydrogen peroxide, SUMOylation was increased. Neither 10% ethanol or hypoxia treatment showed any major ability to increase SUMOylated proteins. **(B)** Heat shock or a mixture of MG132 and PR619 increase SUMOylation in mpkDCT-SUMO1^T95K^ cells.

mpkDCT-SUMO1^T95K^ and mpkDCT-SUMO2^T90K^ cells were cultured as described (Wu et al., 2018). Successful transfection and detection of SUMOylation was determined by stressing cells with: 1) 10 or 20 µM MG132 (Sigma-Aldrich) for 7 h (Hendriks et al., 2014) or 80 min (Castoralova et al., 2012), respectively; 2) 20 µM PR619 (Abcam) for 7 h (Hendriks et al., 2014); 3) 100 mM H_2_O_2_ for 20 min (Bossis and Melchior, 2006); 4) hypoxia for 6 h (restricted O_2_ supply by closing culture flasks); 5) 10% ethanol for 1 h (Bossis and Melchior, 2006); or 6) 43°C heat for 1 h (Castoralova et al., 2012; Hendriks et al., 2014) (Figure 1). For further SUMOylation profiling experiments, cells were treated with either 10 µM MG132 for five hours, with the last hour also containing 22 µM PR619 or with heat shock as above described in parallel experiments. Data analysis was done from the combined data search results. For aldosterone experiments, cells were incubated in pure DMEM-F12 media for 48 h (media changed every 24 h) and subsequently treated with aldosterone (Sigma) in pure DMEM-F12 media. Cells were lysed in Laemmli SDS sample buffer with 15 mg/ml 1,4-Dithiothreitol, sonicated with an ultrasonic homogenizer (150 V/T, BioLogics), and heated for 10 min at 65°C prior to western blotting.

### Apical Surface Biotinylation Assay

mpkDCT-Pept2 and mpkDCT-Pept2^K139R^ cells were cultured on semi-permeable supports until confluent and stable transepithelial electrical resistance was achieved (Wu et al., 2018). Cells were incubated in pure DMEM-F12 media for 48 h and subsequently treated with 1 µM aldosterone or vehicle (DMSO) for 48 h, with media changed every 24 h. Cell surface biotinylation was performed as described (Rosenbaek et al., 2014), except the cell lysis buffer contained the irreversible proteasomal deubiquitylase (DUB) inhibitors N-Ethylmaleimide (2.5 mg/ml, SigmaAldrich) and the general deSUMOylation inhibitor PR619 (22 µM, Abcam).

### Western Blotting

Standard protocols for sample preparation and SDS-PAGE were used, with proteins transferred onto PVDF membranes by electrophoresis (Bio-Rad). Primary antibodies utilized were against SUMO1 (cat# AM1200a, Abgent at 1:250 dilution), SUMO2 (cat# AP1223a, Abgent at 1:250 dilution), FLAG-tag (cat# F7425, SigmaAldrich at 1:1,000 dilution), Actin (cat# A2066, SigmaAldrich at 1:3,000 dilution), Proteasome 20s (cat# sc-67339, Santa Cruz Biotechnology at 1:1,000 dilution) and αENaC at 1:1,000 dilution (Sorensen et al., 2013) (developed and donated kindly by Prof. Johannes Loffing, University of Zurich). Antibodies were diluted in PBS (2.8 mM NaH_2_PO_4_, 4.2 mM Na_2_HPO_4_, 150 mM NaCl, pH 7.4) with 0.1% Tween, 1% BSA and 2 mM NaN_3_ and incubated overnight at 4°C. Immunoreactivity was detected using enhanced chemiluminescence after probing with horseradish peroxidase-conjugated secondary antibodies against rabbit (DAKO P448, goat anti-rabbit IgG) or mouse (DAKO P447, goat anti-mouse IgG) immunoglobulins at 1:5,000 dilution in PBS with 5% skimmed milk for 1 h at room temperature. Images were acquired with an ImageQuant LAS4000 (GE Healthcare) and quantified using Image Studio Lite v5.2 (LI-COR). GraphPad Prism v7 was used for statistical analysis and where appropriate to analyze variances. Statistical tests are listed in the individual figure legends and a *p* value <0.05 were considered as significant.

### Immunohistochemistry

Serial sections of 3 µm thickness were cut from archived FFPE mouse kidney blocks using a rotary microtome (Leica Microsystems) and immunolabelled as previously described (Poulsen et al., 2018) using antibodies against NCC (mouse-monoclonal at 1:5,000 dilution as described in Kortenoeven et al. (2021)) and Pept2 (cat# LS-C357578 and LS-C490072, LS Bio at 1:500 dilution). Immunolabeling was visualized using horseradish peroxidase-conjugated secondary antibodies (DAKO P448, anti-rabbit or DAKO P447, anti-mouse at 1:200 dilution). The antibodies where diluted in PBS, pH 7.4 with 0.5% BSA and 0.05% saponin and incubated at 4°C overnight for primary antibodies and at room temperature for 1 h for the secondary antibodies. The protocol for dual labeling has been previously described (Fenton et al., 2007). Images were taken using a Leica DMRE light microscope equipped with a digital camera (Leica, Wetzlar, Germany).

### Affinity Purification of SUMOylated Proteins Using Ni^2+^-Coated Agarose Beads

The protocol has previously been described in detail (Wu et al., 2019). Briefly, cells were cultured in four T175 flasks to yield enough crude protein lysates for affinity purification. Cell-lysis was performed in customized lysis buffer (8 M urea, 2 M thio-urea, 100 mM sodium phosphate (pH 8), 10 mM Tris (pH 8), 10 mM imidazole, 0.1% SDS, 1% HALT Protease inhibitor (ThermoFischer) and 22 µM PR619). Approximately 60–90 mg of protein lysate was incubated with 800 µl of HisPur™ Ni-NTA Resin (ThermoFischer) in a 10 ml spin column overnight (maximum 18 h) at 4°C with rotation. Columns were washed three times in wash buffer 1 (8 M Urea, 2 M Thio-Urea, 100 mM Sodium Phosphate (pH 8), 10 mM Tris (pH 8), 10 mM Imidazole and 2.5 mM β-Mercaptoethanol (B-ME)), twice in wash buffer 2 (8 M Urea, 2 M Thio-Urea, 100 mM Sodium Phosphate (pH 6.3), 10 mM Tris (pH 8), 10 mM Imidazole and 2.5 mM B-ME) and finally two washes again in wash buffer 1. Elution of the proteins was performed using successive incubations for 20 min with 1 ml elution buffer (8 M Urea, 2 M Thio-Urea, 100 mM Sodium Phosphate (pH 6.3), 10 mM Tris (pH 8) and 400 mM Imidazole). Eluates were combined before subsequent processing.

### Filter-Aided Sample Preparation

Ni-NTA purified proteins were transferred onto Vivacon 500 spin columns (30 kDa cutoff, Sartorius), and washed three times with 400 µl UA buffer (8M urea plus 100 mM triethylammonium bicarbonate). Proteins were incubated with 50 mM dithiothreitol in UA buffer for 1 h at 37°C followed by incubation with 50 mM 2-chloroacetamide in UA buffer in the dark for 20 min at 25°C. Following centrifugation, filters were washed with immunoaffinity purification (IAP) buffer (50 mM MOPS/NaOH pH 7.2 plus 10 mM Na2HPO4 plus 50 mM NaCl) and proteins digested overnight at 37°C with 1:50 lysyl endopeptidase (Lys-C, Wako Pure Chemical Corporation) in IAP buffer. Peptides were collected by centrifugation (Lys-C digested peptides) and the remaining larger peptides on the filters were subjected to digestion with 1:100 glycyl endopeptidase (Glu-C, ThermoFisher) at 25°C overnight (Lys-C + Glu-C digested peptides).

### K-ε-GG Immunoprecipitation (K-ε-GG - IP)

Peptides were purified using the ubiquitin remnant motif (K-ε-GG) kit (Cell Signaling Technology) as described (Tammsalu et al., 2015). Briefly, 3 µl of packed resin was added to 250 µg peptides, the beads were washed three times with IAP buffer and then incubated overnight at 4°C with gentle mixing. Beads were pelleted by centrifugation at 1,000 g and washed several times with 300 µl ice-cold IAP buffer. Finally, the antibody-purified peptides were released from the beads by two 10-min incubations in 150 µl of 0.15% (v/v) trifluoroacetic acid. The eluate was vacuum dried before LC-MS/MS analysis.

### Nano-Liquid Chromatography and Tandem Mass Spectrometry and Data Analysis

Purified peptides were subjected to LC (easy nLC-1000) - MS/MS (QExactive) analysis. A 75-min separation window, with a linear 5–22% of acetonitrile in 0.1% formic acid over 60 min followed by a linear 22–40% of acetonitrile in 0.1% formic acid over 15 min, was used to separate the peptides. The Q-Exactive was operated with data-dependent acquisition mode under an ultra-sensitive setting: MS1 resolution of 70,000, Automatic Gain Control (AGC) target of 1e6, maximum Injection Time (IT) of 20 ms; MS2 resolution of 35,000, loop count of one, AGC target of 5e5 and a maximum IT of 1,000 ms. HCD collision energy was set at 30%. Dynamic exclusion was set at 30 s, and precursor ions with charge state unknown, +1 and above +8 were excluded for fragmentation. The data was analysed by MaxQuant (version 2.0.1.0). All the raw files from one SUMO family (SUMO1 or SUMO2) were searched together against the mouse Uniprot database (19th of July 2021, only the “reviewed” part), with Lys-C/P as enzyme for group 0 (Lys-C digested peptides) and LysC/P plus GluC for group 1 (Lys-C, followed by GluC digested peptides). Acetylation of protein N-term, oxidation of methionine, phosphorylation of serine, threonine and tyrosine, and di-glycine modification of lysine were set as variable modifications, while carbamidomethylation of cysteine was set as a fixed modification. All other parameters were set as default. The mass spectrometry proteomics data have been deposited to the ProteomeXchange Consortium via the PRIDE partner repository (Perez-Riverol et al., 2019) with the dataset identifier PXD028507.

### Bioinformatics

#### Identification

**FIGURE 2:**
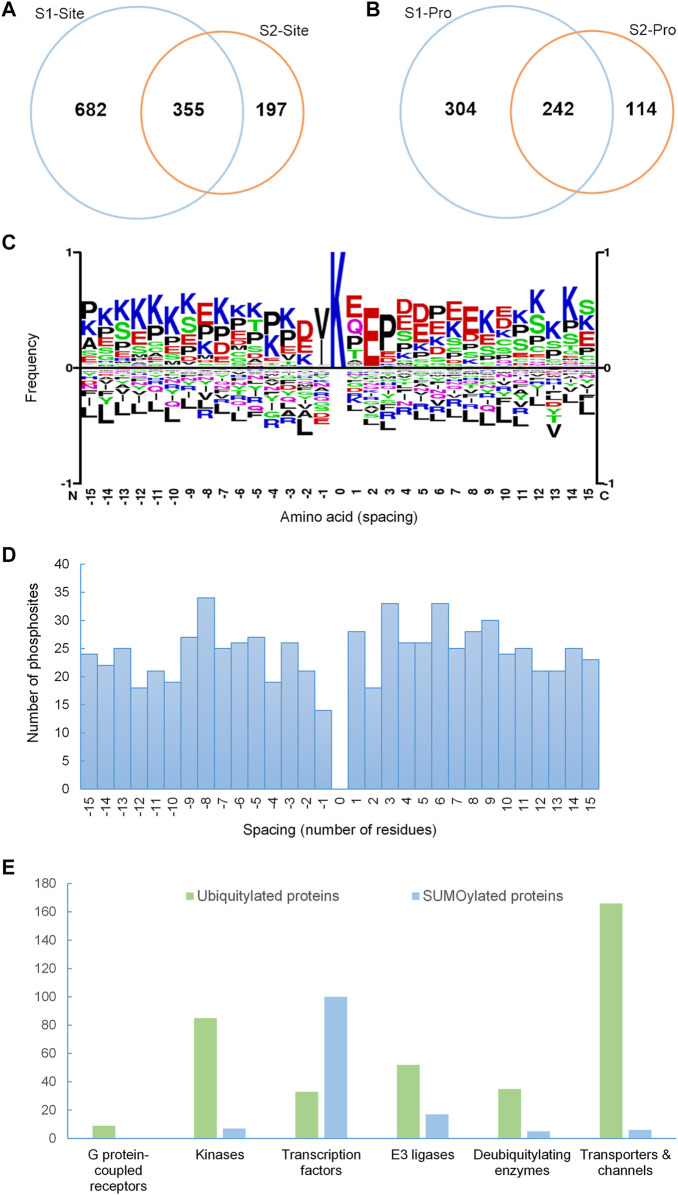
SUMOylation proteome of mpkDCT cells. **(A)** Venn diagram of SUMO1 and SUMO2 sites and **(B)** SUMO1 and SUMO2 proteins from mpkDCT cells. **(C)** Motif analysis revealed a classic KxE consensus motif for the acceptor lysine site and the reversed KxD/E motif. **(D)** Distribution overview of SUMO-phosphorylation occurrences. SUMOylated sites were from this study, and phosphorylation sites were retrieved from PhosphoSitePlus. SUMOylated lysines are at position 0, with spacing indicating the relative position of phosphorylation. **(E)** Comparison of different protein classes between ubiquitylated proteins from mouse kidney and the SUMOylation proteome of mpkDCT cells highlights the high degree of involvement of SUMOylation in gene transcription.

Only SUMO sites with a site probability score (reported by MaxQuant) of above 0.9 were retained for all further analysis (Figures 2A,B).

#### Motif Analysis

Motif analysis was done by SequenceLogo (https://www.phosphosite.org/sequenceLogoAction) (Figure 2C). Input was the 31 amino acids sequence window (+15 and −15 aa with K in the center) surrounding all identified SUMO sites (SUMO1 and SUMO2 combined).

#### Spacing Analysis

Spacing between identified SUMO sites from this study and all known phosphorylation sites (PhosphoSitePlus, https://www.phosphosite.org/homeAction) in close proximity to the SUMO sites was calculated (Figure 2D).

#### Databases

G protein-coupled receptor—Ligand Association Database was retrived from https://zhanglab.ccmb.med.umich.edu/GLASS/ (Chan et al., 2015; Figure 2E). Transporter/channels, protein kinases and transcription factor databases used were retrieved from https://helixweb.nih.gov/ESBL/Database/NephronRNAseq/ (Lee et al., 2015a). The E3 ligase database was retrieved from https://hpcwebapps.cit.nih.gov/ESBL/Database/E3-ligases/ (Medvar et al., 2016). The deubiquitylating enzyme database was retrieved from https://hpcwebapps.cit.nih.gov/ESBL/Database/DUBs/.

#### ClueGO Analysis

**FIGURE 3:**
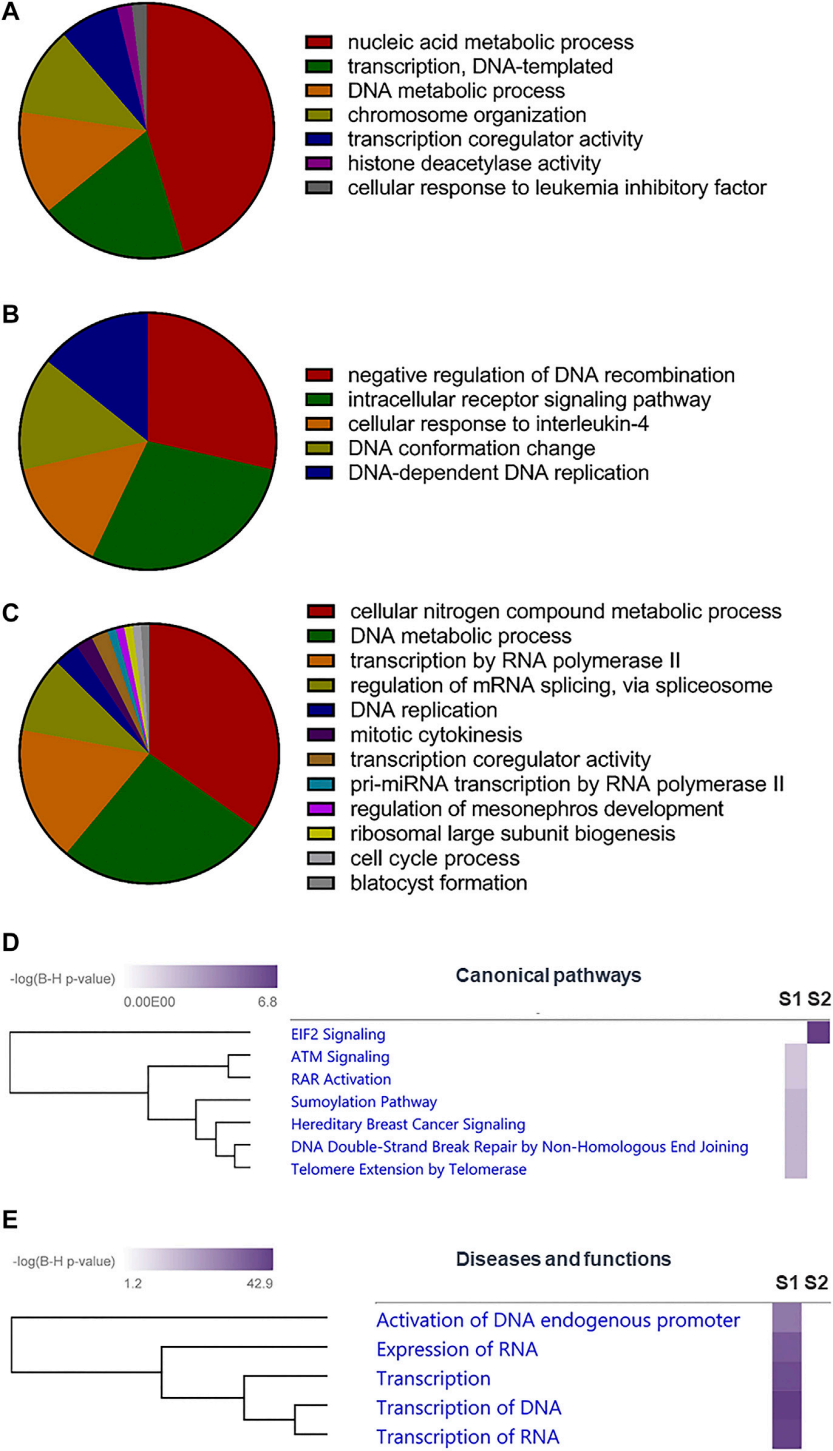
Role of SUMOylation in mpkDCT cells. **(A)** Gene ontology (GO) analysis by ClueGO for SUMO1 and **(B)** SUMO2 exclusive proteins, as well as **(C)** proteins modified by both SUMO1 and SUMO2 in mpkDCT cells. GO analysis highlighted the different roles of SUMO1 and SUMO2. **(D)** Ingenuity Pathway Analysis (IPA) canonical pathway analysis of SUMO1 and SUMO2 exclusive proteins highlighted the high degree of involvement of SUMO2 in Eukaryotic Initiation Factor 2 (EIF2) signalling pathway. **(E)** IPA diseases and functions analysis reaffirmed the importance of SUMOylation in gene transcription.

SUMO1 and SUMO2 unique and shared proteins were assessed using Clue GO (v2.5.8) within the Cytoscape framework (v3.7.2) (Figures 3A–C). Biological process annotations were updated on 29th of July 2021 and used in all circumstances. Evidence level was set at “All Experimental.” “Use GO term fusion” function was enabled and only pathways with a *p* < 0.05 were retained.

#### Ingenuity Pathway Analysis

SUMO1 and SUMO2 unique proteins were assessed individually using core analysis with IPA default parameters (Figures 3D,E). The two core analysis results were combined in a comparison analysis to generate heatmaps for canonical pathways (Log10 Benjamini-Hochberg *p* value cutoff of 2 with hierarchical clustering), as well as diseases and functions (Log10 Benjamini-Hochberg *p* value cutoff of 20 with hierarchical clustering).

#### STRING Analysis

Protein-protein interactions between SUMOylated transcription factors (TFs) and known water and electrolyte transporters/channels (Lee et al., 2015a) were evaluated using STRING (Szklarczyk et al., 2015; version 10, https://string-db.org/; Supplementary Figure S1). Organism was set as *Mus musculus* while all other parameters were left as default. Interactions with high confidence (interaction score ≥0.7) are shown in Supplementary Figure S1.

## Results

### Protein SUMOylation can be Modulated in mpkDCT Cells

Protein SUMOylation is increased by various cellular stresses or inhibition of deSUMOylation pathways (Zhou et al., 2004; Bossis and Melchior, 2006; Tempe et al., 2008; Castoralova et al., 2012; Hendriks et al., 2014). Compared to mpkDCT-wild type (WT) cells (Figure 1A), mpkDCT-SUMO1^T95K^ transfected cells (similar results with mpkDCT-SUMO2^T90K^ cells, see Supplementary Figure S2) displayed large increases in the free SUMO moiety of 15–20 kDa and a broad smear representing a range of SUMOylated proteins after inhibition of deSUMOylation enzymes with PR619 or MG132, or inducing cell stress with hydrogen peroxide. Neither 10% ethanol or hypoxia treatment showed any major ability to increase SUMOylated proteins. Heat shock or a combination of 5 h with MG132 and 1 h with PR619 increased SUMOylation to the highest degree (Figure 1B), therefore both these conditions were used to map the SUMO1 and SUMO2 landscape in mpkDCT cells.

### SUMO1 and SUMO2 Proteome of mpkDCT Cells

1037 SUMO1 and 552 SUMO2 sites, corresponding to 546 SUMO1 and 356 SUMO2 proteins were identified in mpkDCT-SUMO1^T95K^ and mpkDCT-SUMO2^T90K^ cells (Supplementary Table S1). Approximately 29% of SUMOylated sites and 37% of SUMOylated proteins are targeted by both SUMO1 and SUMO2 (Figures 2A,B). However, the majority of SUMOylated proteins had only one SUMO site identified. A further comparison with our previous study on collecting duct principal cells (Wu et al., 2019) revealed a considerable amount of overlap of SUMOylation events between collecting duct and DCT cells (Supplementary Figure S3). Most SUMOylated sites fitted a classic KxE consensus motif, but a reversed KxD/E motif was also revealed by motif analysis (Figure 2C). The SUMO-phosphorylation co-modification scenario (presence of both SUMOylation and phosphorylation within a short amino acid sequence) was assessed by taking all known phosphorylation sites (PhosphoSitePlus, https://www.phosphosite.org/homeAction) into consideration (Figure 2D). Phosphorylation events surrounding SUMOylation sites were roughly distributed evenly. Similar scenario was observed in other studies (Hendriks et al., 2017; Hendriks et al., 2018; Wu et al., 2019) and may indicate that SUMOylation is partially dependent on phosphorylation events, and a moderate spacing between SUMOylation and phosphorylation may facilitate SUMOylation (Su et al., 2012; Khan et al., 2014; Tomasi and Ramani, 2018; Uzoma et al., 2018). Despite a considerably smaller SUMOylation proteome (660 SUMOylated proteins) compared to a mouse kidney ubiquitylation database (2,725 ubiquitylated proteins), more transcription factors (TFs) were SUMOylated than ubiquitylated, with approximately 15% of the mpkDCT SUMO proteome being TFs whereas only 1.2% of the mouse kidney ubiquitylome were TFs (Figure 2E). All other categories analyzed, including GPCRs, protein kinases, E3 ubiquitin ligases, deubiquitylating enzymes, and transporters and channels follow the opposite trend and are in proportion to the number of proteins in their respective proteome.

### Role of SUMOylated Proteins in mpkDCT Cells

Gene ontology (GO) analysis suggested that proteins in mpkDCT cells SUMOylated by SUMO1 and SUMO2 were associated with different cellular functions. Proteins SUMOylated by only SUMO1 were predominantly associated with nucleic acid metabolic process (Figure 3A), whereas those targeted by only SUMO2 were enriched for negative regulation of DNA recombination and intracellular receptor signaling pathways (Figure 3B). Proteins that were SUMOylated by both SUMO1 and SUMO2 were associated with various metabolic processes and transcription-related functions (Figure 3C). Ingenuity Pathway Analysis (IPA) canonical pathway analysis highlighted the high-degree of involvement of SUMO2 proteins in the Eukaryotic Initiation Factor 2 (EIF2) signaling pathway (Figure 3D). IPA diseases and functions analysis reaffirmed the involvement of SUMOylation in DNA/RNA processing and transcription (Rosonina et al., 2017; Boulanger et al., 2021) (Figure 3E), in line with the large number of transcription factors being SUMOylated (Figure 2E). Many of the identified SUMOylated TF genes were suggested to strongly interact with important water and electrolyte/solute transporters and channels by STRING analysis (Supplementary Figure S1). These include Gata3 and Nfat5 (high confidence for regulating the water channel AQP2), Ncor1 and Ncor2 (high confidence for regulating the glucose transporter GLUT4), and Ehf (high confidence for regulating the tight junction protein Claudin 7).

### Renal Hydrogen-Coupled Oligopeptide and Drug Co-Transporter Pept2 is Expressed in the Distal Convoluted Tubule in Mouse Kidney

**FIGURE 4:**
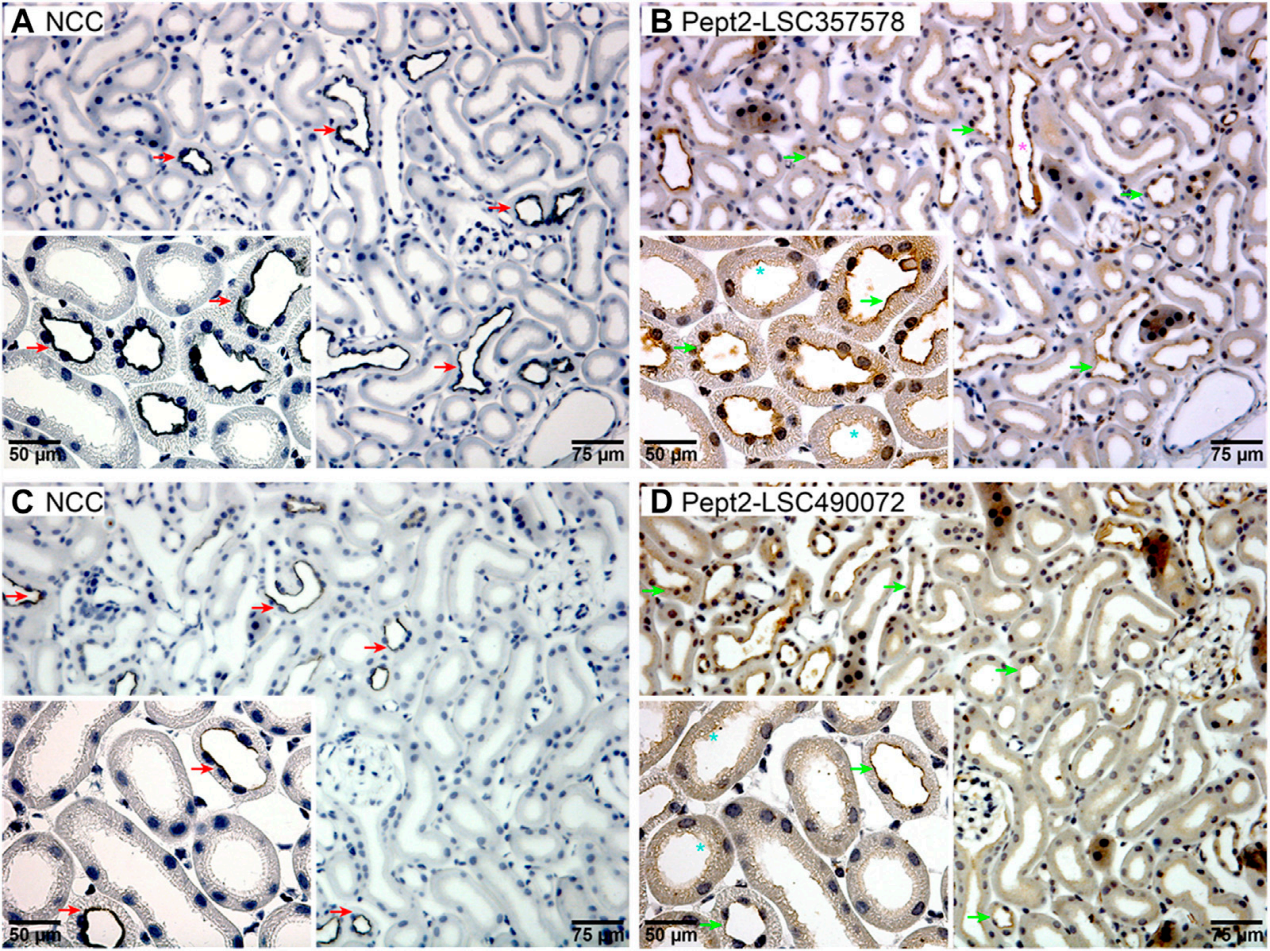
Pept2 is expressed in the apical membrane of mouse DCT. Serial sections from mouse kidney were probed with an NCC antibody **(A,C)** to identify the DCT. Two different Pept2 antibodies **(B,D)** showed Pept2 in the apical membrane domain of the same segment as NCC as highlighted by the red and green arrows. Turquoise stars (*) mark proximal tubules where Pept2 is also detected in the apical brush border. Pink star (*) represents Pept2 labeling of the collecting duct. Magnified inserts are shown in left-hand corners.

Our interest lies within the role of SUMOylation for modulating renal membrane transporters. In mpkDCT cells we identified six SUMOylated membrane transporters (Supplementary Table S1). One of them, Pept2, was identified in our previous study to be SUMOyated at the same K139 site and increased in response to aldosterone in mouse kidney cortical collecting duct cells (Wu et al., 2019). Pept2 is classically described as a proximal tubule brush border localized high-affinity/low-capacity transporter that is important for transporting and maintaining metabolic stability of oligopeptide-like drugs such as beta-lactam antibiotics, antivirals, angiotensin converting enzyme inhibitors and oncological drugs (Liu et al., 1995; Shen et al., 1999; Daniel and Rubio-Aliaga, 2003; Daniel and Kottra, 2004; Luckner and Brandsch, 2005). To confirm it is also expressed in the DCT *in vivo*, we performed immunolabelling of serial sections from mouse kidney using an antibody against the DCT-specific NaCl cotransporter NCC and two different antibodies against Pept2 (Figure 4). In addition to labeling of the proximal tubule brush border, Pept2 immunoreactivity was also relatively abundant in the apical membrane domain of NCC positive tubules, indicating Pept2 is expressed in the DCT. Pept2 labelling was also observed in tubules morphologically resembling collecting ducts, supporting our previous identification of SUMOylated Pept2 in cells from this segment (Wu et al., 2019).

### SUMOylation of K139 Plays a Role in Membrane Localization of Pept2

**FIGURE 5:**
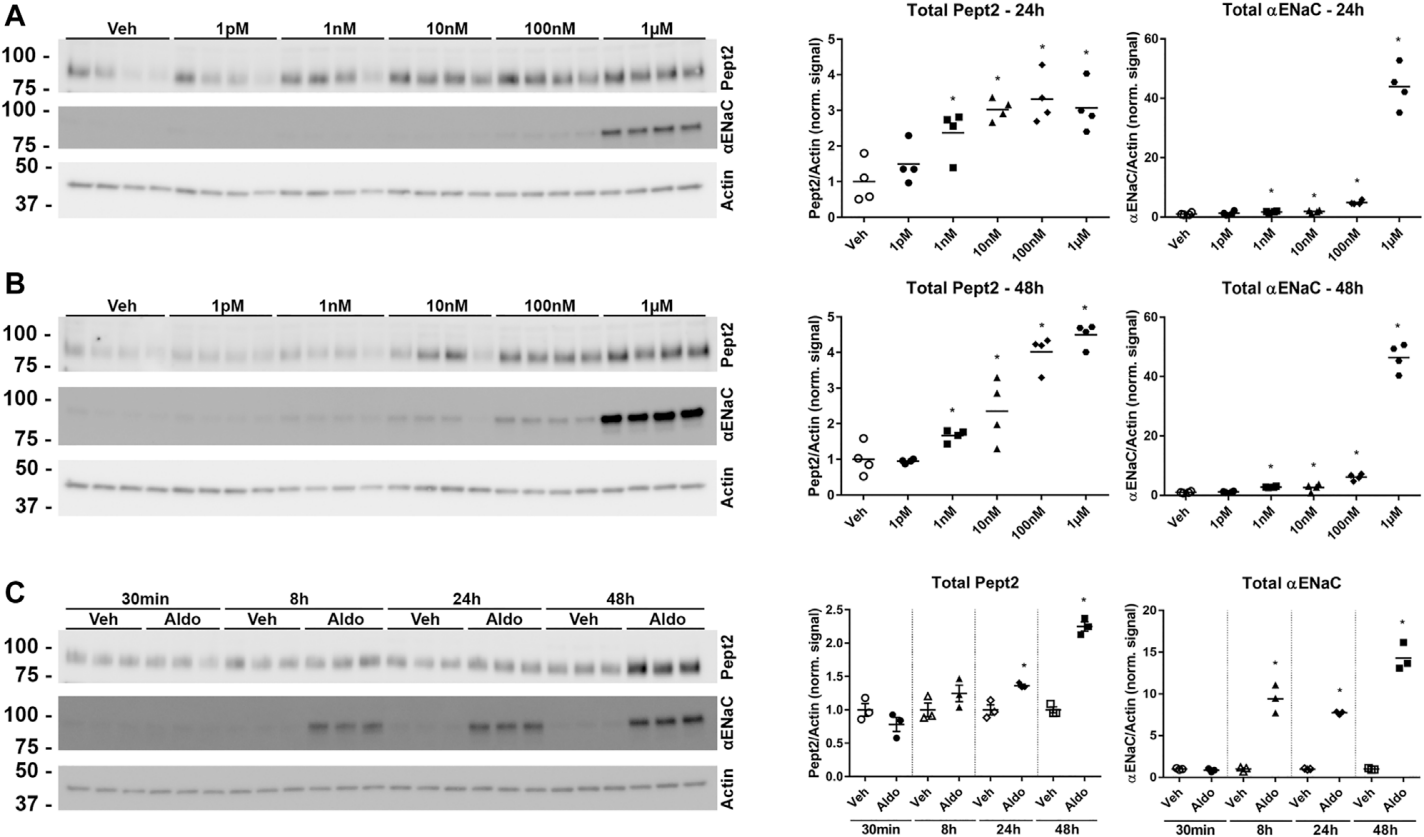
Pept2 is an aldosterone induced protein. **(A)** mpkDCT cells stably transfected with wild-type mouse FLAG-tagged-Pept2 (WT Pept2) were treated with vehicle (DMSO) or increasing concentrations of aldosterone for 24 h. Samples were probed with antibodies against actin, αENaC and FLAG (detecting Pept2). **(B)** Similar experiment but 48 h treatment. **(C)** Increases in Pept2 in mpkDCT cells in response to 1 µM aldosterone over time. *p < 0.05 in unpaired *t*-test versus vehicle treatment of same cell line.

**FIGURE 6:**
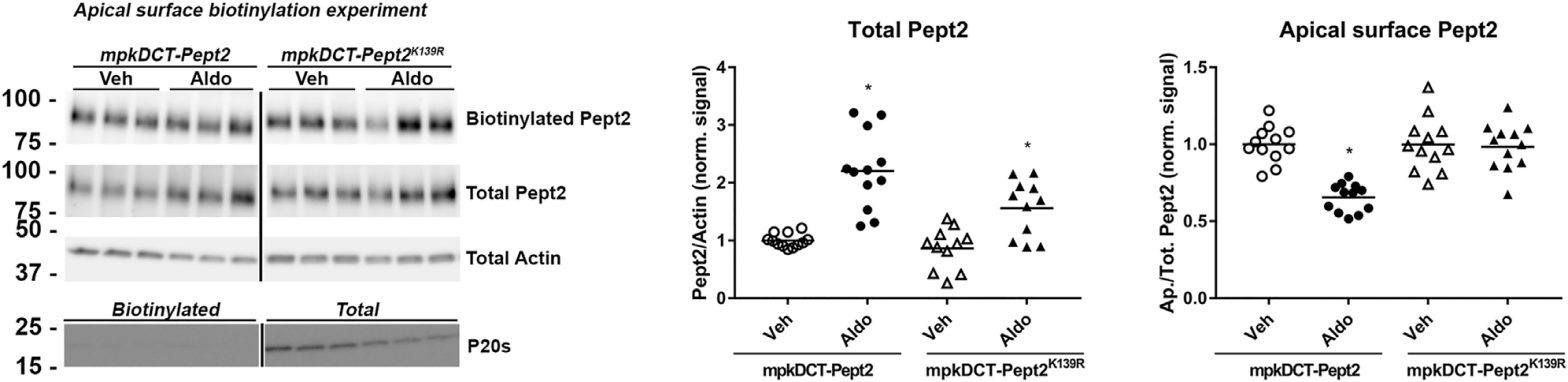
Role of K139 SUMOylation of Pept2 in membrane targeting upon aldosterone treatment. mpkDCT-Pept2 and mpkDCT-Pept2^K139R^ expressing cells were treated with 1 µM aldosterone for 48 h and subjected to apical surface biotinylation. Samples were probed with anti-FLAG (Pept2), actin and proteosome 20s antibodies. *p < 0.05 in unpaired *t*-test versus vehicle treatment of same cell line.

To investigate further the role of K139 in Pept2, we generated mpkDCT cells expressing FLAG-tagged Pept2-WT (mpkDCT-Pept2) or a K139R mutant that prevents this site being SUMOylated (mpkDCT-Pept2^K139R^). The FLAG-tag was necessary since, despite their applicability to immunolabeling, the Pept2 antibodies we tested were unsuitable for western blotting. In Pept2-WT cells, concentrations of aldosterone >1 nM significantly increased total Pept2 two- to four-fold after 24 h or 48 h treatment (Figures 5A,B respectively). Even at the highest dose (1 µM), aldosterone effects on Pept2 abundance were not observed until 24 h of treatment, whereas effects on another aldosterone-induced protein the α-subunit of the epithelial sodium channel ENAC were observed after 8 h (Figure 5C). *In vivo*, Pept2 was localized to the apical membrane of DCT cells (Figure 4). To investigate the role of aldosterone for altering the membrane localization of Pept2 and a potential role of SUMOylation at K139, mpkDCT-Pept2 and mpkDCT-Pept2^K139R^ cells were treated with aldosterone (1 µM) for 48 h and apical surface biotinylation performed. Aldosterone increased total Pept2 abundance in both mpkDCT-Pept2 and mpkDCT-Pept2^K139R^ cells (Figure 6). In mpkDCT-Pept2 cells, aldosterone decreased the fraction of Pept2 (relative to total cellular amount) on the apical plasma membrane, suggesting that upon exposure to aldosterone Pept2 is either internalized or has reduced trafficking to the apical membrane. In contrast, in mpkDCT-Pept2^K139R^ cells, Pept2 membrane levels were not altered by aldosterone, suggesting that SUMOylation of K139 is involved in the regulated trafficking of the co-transporter.

## Discussion

Protein SUMOylation allows a diverse response of cells to various forms of stress. As kidney epithelial cells are subjected to genotoxic, osmotic, and oxidative stresses, it is therefore not surprising that protein SUMOylation increases in various forms of kidney disease (Guo et al., 2015; Dong et al., 2016; Li et al., 2019). However, protein SUMOylation remains a relatively under investigated form of protein post-translational modification and the role of SUMOylation in different kidney epithelial cells remains unclear. Here, we generated a SUMOylation landscape of renal DCT cells as a first step to understanding the role of SUMOylation in this specific cell type. Furthermore, we demonstrate that SUMOylation of a specific transport protein, Pept2, is important for modulating the function of this protein after aldosterone stimulation, suggesting that alterations in protein SUMOylation may play a role under hormone stimulated conditions.

To generate a SUMOylation proteome of DCT cells we used the approach described initially by Tammsalu et al. (2015) and adapted by us to study collecting duct cells (Wu et al., 2019). This approach introduces single amino acid substitutions into SUMO, which upon overexpression in cells allows the addition of this PTM to target proteins to be differentiated from other similar PTMs, including ubiquitylation and neddylation. SUMO is an extremely low abundant modification under normal physiological conditions and severe cellular stresses are required to increase its attachment to target proteins (Hendriks et al., 2014). Our aim here was to induce as much SUMOylation as possible to generate a comprehensive SUMOylation proteome of DCT cells. Therefore, we adopted multiple treatments from other studies, including MG132 and PR619, as well as heat shock, and combined all the data into one single search (Hendriks et al., 2014; Hendriks et al., 2017). Analysis of the SUMO proteome of mpkDCT-SUMO1^T95K^ and mpkDCT-SUMO2^T90K^ cells highlighted overlap between SUMO1 and SUMO2 sites (29%) and SUMOylated proteins (37%), emphasizing substrate cross-reactivity between the isoforms. Combining the current data with those from mouse renal cortical collecting duct (mpkCCD) cells, a total of 2811 and 1,260 SUMOylation sites and proteins respectively have been identified in renal epithelial cells, suggesting that protein SUMOylation is a relatively frequent occurrence in epithelial cells. Not surprisingly for two neighbouring tubule sections with some shared functional characteristics (Wu et al., 2018), there was also a considerable overlap between mpkCCD and mpkDCT cell lines, accounting for 25 and 37% of total SUMOylation sites and proteins respectively. Although some sites and proteins were highly conserved between the cell types (Supplementary Figure S3), much less SUMO2 modified sites and proteins were identified in DCT cells relative to collecting duct cells. Whether this is a reality *in vivo* or a technical limitation of this approach is unclear. Importantly, the number of SUMOylation sites and proteins identified are in line with the original study using this approach (Hendriks et al., 2018), but this number could likely be increased with greater technical replicates and peptide fractionation. Other recently developed techniques have been proven to be more efficient for identifying SUMOylated proteins, but the approaches used are less physiological with many mutations and the use of suspension grown carcinoma cells (Hendriks et al., 2014; Hendriks et al., 2017). However, over 14,000 endogenous SUMO2 sites were identified in human cells and mouse organs following a peptide level antibody enrichment technique (Hendriks et al., 2018). Application of this particular technique in future projects could be a powerful tool to elucidate the modulation of SUMOylated proteins *in vivo* in various disease models, including kidney disease.

Large number of phosphorylation sites are in close proximity to the identified SUMO sites, and this suggests that co-modification might be important for either of the PTMs to occur. A phosphorylation-dependent SUMO modification (PDSM) motif, composed of a SUMO consensus site and an adjacent proline-directed phosphorylation site (ΨKxExxSP), promotes SUMOylation is apparent in several regulators of gene expression, including heat shock factors (HSFs) and TFs (Hietakangas et al., 2006). In contrast to ubiquitylation, a modification with many known functions (Dittmar and Winklhofer, 2019; Deng et al., 2020; Zhu et al., 2020; Mirsanaye et al., 2021), the substantially greater number of SUMOylated TFs highlight the importance of SUMOylation for modulating DNA transcription. SUMOylation modulates the function of TFs through multiple mechanisms, including interaction with histone deacetylase complexes and other transcriptional coregulators, inhibition of acetylation and phosphorylation, as well as availability to bind chromatin (Rosonina et al., 2017). There was also a high correlation (STRING interaction score ≥0.7 with default parameters) between SUMOylated TFs and several important membrane transport proteins in the kidney, including the water channel AQP2, the urea transporter UT-A2, and the glucose transporter GLUT4 (Supplementary Figure S1), suggesting that SUMOylation of TFs may play an important role for controlling their gene expression.

Pept2 (encoded by *Slc15a2*) was one of six SUMOylated membrane transport proteins identified in mpkDCT cells (Supplementary Table S1). Pept2 has previously been localized to the apical compartment of S2 and S3 segments of the proximal tubule (Smith et al., 1998; Shen et al., 1999), where as a member of the family of proton-coupled peptide transporters it plays an important role in absorption of small peptides, as well as beta-lactam antibiotics and other drugs (Daniel and Kottra, 2004; Launay-Vacher et al., 2006). Here we identified Pept2 in mpkDCT cells *in vitro* and in mouse DCT *in vivo*, supporting its presence in this cell type as inferred from recent proteomic and transcriptomic studies (Lee et al., 2015a; Limbutara et al., 2020). The abundance of Pept2 was increased by the mineralocorticoid aldosterone, suggesting that it has a physiological role in the DCT. The physiological role of SUMOylation in Pept2 function was further supported by the lack of altered Pept2 plasma membrane localization after aldosterone treatment in cells expressing a K139R mutant form of Pept2 that cannot be SUMOylated. Technical limitations prevented us from examining the SUMOylation status of Pept2 *in vivo*. Interestingly, patients with specific mutations in SLC15A2 (encoding Pept2) have a longer progression-free survival against hepatocellular carcinoma when treated with sorafenib (Lee et al., 2015b; Minhas and Newstead, 2019). These effects are attributed to increased stability of Pept2 due to alterations in the phosphorylation status of Pept2 and altered drug transport capacity. Studies of whether SUMOylation of Pept2 also alters its stability or plays a role in drug transport would be informative.

In conclusion, this study generated a SUMOylation landscape of mpkDCT cells and determined that the function of the drug transporter Pept2 can be regulated by SUMOylation on a specific site. Our results indicate that protein modification by SUMOylation is a mechanism within renal epithelial cells to modulate gene transcription under various physiological or pathophysiological conditions, but it may also directly influence the activity of various membrane transport proteins and channels.

## Data Availability

The datasets presented in this study can be found in online repositories. The names of the repository/repositories and accession number(s) can be found in the article/Supplementary Material.

## References

[B1] Becker J. Barysch S. V. Karaca S. Dittner C. Hsiao H.-H. Diaz M. B. (2013). Detecting Endogenous SUMO Targets in Mammalian Cells and Tissues. Nat. Struct. Mol. Biol. 20 (4), 525–531. 10.1038/nsmb.2526 23503365

[B2] Bohren K. M. Nadkarni V. Song J. H. Gabbay K. H. Owerbach D. (2004). A M55V Polymorphism in a Novel SUMO Gene (SUMO-4) Differentially Activates Heat Shock Transcription Factors and is Associated With Susceptibility to Type I Diabetes Mellitus. J. Biol. Chem. 279 (26), 27233–27238. 10.1074/jbc.M402273200 15123604

[B3] Bossis G. Melchior F. (2006). Regulation of SUMOylation by Reversible Oxidation of SUMO Conjugating Enzymes. Mol. Cell 21 (3), 349–357. 10.1016/j.molcel.2005.12.019 16455490

[B4] Boulanger M. Chakraborty M. Tempé D. Piechaczyk M. Bossis G. (2021). SUMO and Transcriptional Regulation: The Lessons of Large-Scale Proteomic, Modifomic and Genomic Studies. Molecules 26 (4), 828. 10.3390/molecules26040828 33562565 PMC7915335

[B5] Briet M. Schiffrin E. L. (2010). Aldosterone: Effects on the Kidney and Cardiovascular System. Nat. Rev. Nephrol. 6 (5), 261–273. 10.1038/nrneph.2010.30 20234356

[B6] Častorálová M. Březinová D. Švéda M. Lipov J. Ruml T. Knejzlík Z. (2012). SUMO-2/3 Conjugates Accumulating under Heat Shock or MG132 Treatment Result Largely From New Protein Synthesis. Biochim. Biophys. Acta Mol. Cell Res. 1823 (4), 911–919. 10.1016/j.bbamcr.2012.01.010 22306003

[B7] Chan W. K. B. Zhang H. Yang J. Brender J. R. Hur J. Özgür A. (2015). GLASS: A Comprehensive Database for Experimentally Validated GPCR-Ligand Associations. Bioinformatics 31 (18), 3035–3042. 10.1093/bioinformatics/btv302 25971743 PMC4668776

[B8] Cheng L. Wu Q. Kortenoeven M. L. A. Pisitkun T. Fenton R. A. (2015). A Systems Level Analysis of Vasopressin-Mediated Signaling Networks in Kidney Distal Convoluted Tubule Cells. Sci. Rep. 5, 12829. 10.1038/srep12829 26239621 PMC4523861

[B9] Daniel H. Rubio-Aliaga I. (2003). An Update on Renal Peptide Transporters. Am. J. Physiol. Ren. Physiol. 284 (5), F885–F892. 10.1152/ajprenal.00123.2002 12676733

[B10] Deng L. Meng T. Chen L. Wei W. Wang P. (2020). The Role of Ubiquitination in Tumorigenesis and Targeted Drug Discovery. Sig Transduct. Target. Ther. 5 (1), 11. 10.1038/s41392-020-0107-0 PMC7048745 32296023

[B11] Dittmar G. Winklhofer K. F. (2019). Linear Ubiquitin Chains: Cellular Functions and Strategies for Detection and Quantification. Front. Chem. 7, 915. 10.3389/fchem.2019.00915 31998699 PMC6966713

[B12] Dong B. Gao Y. Kang X. Gao H. Zhang J. Guo H. (2016). SENP1 Promotes Proliferation of Clear Cell Renal Cell Carcinoma through Activation of Glycolysis. Oncotarget 7 (49), 80435–80449. 10.18632/oncotarget.12606 27741516 PMC5348332

[B13] Fenton R. A. Brønd L. Nielsen S. Praetorius J. (2007). Cellular and Subcellular Distribution of the Type-2 Vasopressin Receptor in the Kidney. Am. J. Physiol. Ren. Physiol. 293 (3), F748–F760. 10.1152/ajprenal.00316.2006 17553938

[B14] Flotho A. Melchior F. (2013). Sumoylation: A Regulatory Protein Modification in Health and Disease. Annu. Rev. Biochem. 82, 357–385. 10.1146/annurev-biochem-061909-093311 23746258

[B15] Geiss-Friedlander R. Melchior F. (2007). Concepts in Sumoylation: A Decade On. Nat. Rev. Mol. Cell Biol. 8 (12), 947–956. 10.1038/nrm2293 18000527

[B16] Guo C. Wei Q. Su Y. Dong Z. (2015). SUMOylation Occurs in Acute Kidney Injury and Plays a Cytoprotective Role. Biochim. Biophys. Acta Mol. Basis Dis. 1852 (3), 482–489. 10.1016/j.bbadis.2014.12.013 PMC4386022 25533125

[B17] Hay R. T. (2005). Sumo. Mol. Cell 18 (1), 1–12. 10.1016/j.molcel.2005.03.012 15808504

[B18] Hendriks I. A. D'Souza R. C. J. Yang B. Verlaan-de Vries M. Mann M. Vertegaal A. C. O. (2014). Uncovering Global SUMOylation Signaling Networks in a Site-Specific Manner. Nat. Struct. Mol. Biol. 21 (10), 927–936. 10.1038/nsmb.2890 25218447 PMC4259010

[B19] Hendriks I. A. Lyon D. Su D. Skotte N. H. Daniel J. A. Jensen L. J. (2018). Site-Specific Characterization of Endogenous SUMOylation Across Species and Organs. Nat. Commun. 9 (1), 2456. 10.1038/s41467-018-04957-4 29942033 PMC6018634

[B20] Hendriks I. A. Lyon D. Young C. Jensen L. J. Vertegaal A. C. O. Nielsen M. L. (2017). Site-Specific Mapping of the Human SUMO Proteome Reveals Co-Modification With Phosphorylation. Nat. Struct. Mol. Biol. 24 (3), 325–336. 10.1038/nsmb.3366 28112733

[B21] Hendriks I. A. Vertegaal A. C. O. (2016). A Comprehensive Compilation of SUMO Proteomics. Nat. Rev. Mol. Cell Biol. 17 (9), 581–595. 10.1038/nrm.2016.81 27435506

[B22] Hietakangas V. Anckar J. Blomster H. A. Fujimoto M. Palvimo J. J. Nakai A. (2006). PDSM, a Motif for Phosphorylation-Dependent SUMO Modification. Proc. Natl. Acad. Sci. 103 (1), 45–50. 10.1073/pnas.0503698102 16371476 PMC1324973

[B23] Hoorn E. J. Gritter M. Cuevas C. A. Fenton R. A. (2020). Regulation of the Renal NaCl Cotransporter and its Role in Potassium Homeostasis. Physiol. Rev. 100 (1), 321–356. 10.1152/physrev.00044.2018 31793845

[B24] Hu X. Liu Z. Duan X. Han X. Yuan M. Liu L. (2021). Blocking MCT4 SUMOylation Inhibits the Growth of Breast Cancer Cells. Mol. Carcinog. 60, 702–714. 10.1002/mc.23336 34347919

[B25] Imbert F. Langford D. (2021). Viruses, SUMO, and Immunity: The Interplay Between Viruses and the Host SUMOylation System. J. Neurovirol. 27, 531–541. 10.1007/s13365-021-00995-9 34342851 PMC8330205

[B26] Jiménez-Canino R. Lorenzo-Díaz F. Odermatt A. Bailey M. A. Livingstone D. E. W. Jaisser F. (2017). 11β-HSD2 SUMOylation Modulates Cortisol-Induced Mineralocorticoid Receptor Nuclear Translocation Independently of Effects on Transactivation. Endocrinology 158 (11), 4047–4063. 10.1210/en.2017-00440 28938454

[B27] Ke S. Liu Y.-Y. Karthikraj R. Kannan K. Jiang J. Abe K. (2021). Thyroid Hormone Receptor β Sumoylation Is Required for Thyrotropin Regulation and Thyroid Hormone Production. JCI Insight 6 (16), e149425. 10.1172/jci.insight.149425 PMC8410017 34237030

[B28] Khan M. Rozhon W. Unterholzner S. J. Chen T. Eremina M. Wurzinger B. (2014). Interplay Between Phosphorylation and SUMOylation Events Determines CESTA Protein Fate in Brassinosteroid Signalling. Nat. Commun. 5, 4687. 10.1038/ncomms5687 25134617 PMC4167607

[B29] Kortenoeven M. L. A. Esteva-Font C. Dimke H. Poulsen S. B. Murali S. K. Fenton R. A. (2021). High Dietary Potassium Causes Ubiquitin-Dependent Degradation of the Kidney Sodium-Chloride Cotransporter. J. Biol. Chem. 297 (2), 100915. 10.1016/j.jbc.2021.100915 34174287 PMC8318901

[B30] Kottra G. Daniel H. (2004). The Proton Oligopeptide Cotransporter Family SLC15 in Physiology and Pharmacology. Pflugers Archiv Eur. J. Physiol. 447 (5), 610–618. 10.1007/s00424-003-1101-4 12905028

[B31] Kroonen J. S. Kruisselbrink A. B. Briaire-de Bruijn I. H. Olaofe O. O. Bovée J. V. M. G. Vertegaal A. C. O. (2021). SUMOylation is Associated With Aggressive Behavior in Chondrosarcoma of Bone. Cancers 13 (15), 3823. 10.3390/cancers13153823 34359724 PMC8345166

[B32] Launay-Vacher V. Izzedine H. Karie S. Hulot J. S. Baumelou A. Deray G. (2006). Renal Tubular Drug Transporters. Nephron Physiol. 103 (3), p97–p106. 10.1159/000092212 16554667

[B33] Lee J. W. Chou C.-L. Knepper M. A. (2015a). Deep Sequencing in Microdissected Renal Tubules Identifies Nephron Segment-Specific Transcriptomes. J. Am. Soc. Nephrol. 26 (11), 2669–2677. 10.1681/ASN.2014111067 25817355 PMC4625681

[B34] Lee Y.-S. Kim B. H. Kim B. C. Shin A. Kim J. S. Hong S.-H. (2015b). SLC15A2 Genomic Variation Is Associated with the Extraordinary Response of Sorafenib Treatment: Whole-Genome Analysis in Patients with Hepatocellular Carcinoma. Oncotarget 6 (18), 16449–16460. 10.18632/oncotarget.3758 25965825 PMC4599281

[B35] Li O. Ma Q. Li F. Cai G.-Y. Chen X.-M. Hong Q. (2019). Progress of Small Ubiquitin-Related Modifiers in Kidney Diseases. Chin. Med. J. 132 (4), 466–473. 10.1097/CM9.0000000000000094 30707172 PMC6595721

[B36] Liang Y.-C. Lee C.-C. Yao Y.-L. Lai C.-C. Schmitz M. L. Yang W.-M. (2016). SUMO5, a Novel Poly-SUMO Isoform, Regulates PML Nuclear Bodies. Sci. Rep. 6, 26509. 10.1038/srep26509 27211601 PMC4876461

[B37] Limbutara K. Chou C.-L. Knepper M. A. (2020). Quantitative Proteomics of All 14 Renal Tubule Segments in Rat. J. Am. Soc. Nephrol. 31 (6), 1255–1266. 10.1681/ASN.2020010071 32358040 PMC7269347

[B38] Liu W. Liang R. Ramamoorthy S. Fei Y.-J. Ganapathy M. E. Hediger M. A. (1995). Molecular Cloning of PEPT 2, a New Member of the H+/Peptide Cotransporter Family, from Human Kidney. Biochim. Biophys. Acta Biomemb. 1235 (2), 461–466. 10.1016/0005-2736(95)80036-f 7756356

[B39] Liu Z. Bian X. Gao W. Su J. Ma C. Xiao X. (2021). Rg3 Promotes the SUMOylation of SERCA2a and Corrects Cardiac Dysfunction in Heart Failure. Pharmacol. Res. 172, 105843. 10.1016/j.phrs.2021.105843 34428586

[B40] Luckner P. Brandsch M. (2005). Interaction of 31 β-lactam Antibiotics with the H+/Peptide Symporter PEPT2: Analysis of Affinity Constants and Comparison with PEPT1. Eur. J. Pharm. Biopharm. 59 (1), 17–24. 10.1016/j.ejpb.2004.07.008 15567297

[B41] Ma X. Yang T. Luo Y. Wu L. Jiang Y. Song Z. (2019). TRIM28 Promotes HIV-1 Latency by SUMOylating CDK9 and Inhibiting P-TEFb. Elife 8, e42426. 10.7554/eLife.42426 30652970 PMC6361614

[B42] Ma Y. North B. J. Shu J. (2021). Regulation of Topoisomerase II Stability and Activity by Ubiquitination and SUMOylation: Clinical Implications for Cancer Chemotherapy. Mol. Biol. Rep. 48, 6589–6601. 10.1007/s11033-021-06665-7 34476738 PMC8589089

[B43] Matic I. Schimmel J. Hendriks I. A. van Santen M. A. van de Rijke F. van Dam H. (2010). Site-specific Identification of SUMO-2 Targets in Cells Reveals an Inverted SUMOylation Motif and a Hydrophobic Cluster SUMOylation Motif. Mol. Cell 39 (4), 641–652. 10.1016/j.molcel.2010.07.026 20797634

[B44] Medvar B. Raghuram V. Pisitkun T. Sarkar A. Knepper M. A. (2016). Comprehensive Database of Human E3 Ubiquitin Ligases: Application to Aquaporin-2 Regulation. Physiol. Genom. 48 (7), 502–512. 10.1152/physiolgenomics.00031.2016 PMC4967219 27199454

[B45] Minhas G. S. Newstead S. (2019). Structural Basis for Prodrug Recognition by the SLC15 Family of Proton-Coupled Peptide Transporters. Proc. Natl. Acad. Sci. USA 116 (3), 804–809. 10.1073/pnas.1813715116 30602453 PMC6338836

[B46] Mirsanaye A. S. Typas D. Mailand N. (2021). Ubiquitylation at Stressed Replication Forks: Mechanisms and Functions. Trends Cell Biol. 31 (7), 584–597. 10.1016/j.tcb.2021.01.008 33612353

[B47] Peng C. Tan Y. Yang P. Jin K. Zhang C. Peng W. (2021). Circ-GALNT16 Restrains Colorectal Cancer Progression by Enhancing the SUMOylation of hnRNPK. J. Exp. Clin. Cancer Res. 40 (1), 272. 10.1186/s13046-021-02074-7 34452628 PMC8400830

[B48] Peng K.-C. Cluzeaud F. Bens M. Duong Van Huyen J.-P. Wioland M. A. Lacave R. (1999). Tissue and Cell Distribution of the Multidrug Resistance-Associated Protein (MRP) in Mouse Intestine and Kidney. J. Histochem. Cytochem. 47 (6), 757–767. 10.1177/002215549904700605 10330452

[B49] Perez-Riverol Y. Csordas A. Bai J. Bernal-Llinares M. Hewapathirana S. Kundu D. J. (2019). The PRIDE Database and Related Tools and Resources in 2019: Improving Support for Quantification Data. Nucleic Acids Res. 47 (D1), D442–D450. 10.1093/nar/gky1106 30395289 PMC6323896

[B50] Poulsen S. B. Marin De Evsikova C. Murali S. K. Praetorius J. Chern Y. Fenton R. A. (2018). Adenylyl Cyclase 6 is Required for Maintaining Acid-Base Homeostasis. Clin. Sci. 132 (16), 1779–1796. 10.1042/CS20180060 PMC6512970 29941522

[B51] Rosenbaek L. L. Kortenoeven M. L. A. Aroankins T. S. Fenton R. A. (2014). Phosphorylation Decreases Ubiquitylation of the Thiazide-Sensitive Cotransporter NCC and Subsequent Clathrin-Mediated Endocytosis. J. Biol. Chem. 289 (19), 13347–13361. 10.1074/jbc.M113.543710 24668812 PMC4036343

[B52] Rosonina E. Akhter A. Dou Y. Babu J. Sri Theivakadadcham V. S. (2017). Regulation of Transcription Factors by Sumoylation. Transcription 8 (4), 220–231. 10.1080/21541264.2017.1311829 28379052 PMC5574528

[B53] Sajeev T. K. Joshi G. Arya P. Mahajan V. Chaturvedi A. Mishra R. K. (2021). SUMO and SUMOylation Pathway at the Forefront of Host Immune Response. Front. Cell Dev. Biol. 9, 681057. 10.3389/fcell.2021.681057 34336833 PMC8316833

[B54] Shen H. Smith D. E. Yang T. Huang Y. G. Schnermann J. B. Brosius F. C. 3rd (1999). Localization of PEPT1 and PEPT2 Proton-Coupled Oligopeptide Transporter mRNA and Protein in Rat Kidney. Am. J. Physiol. Ren. Physiol. 276 (5), F658–F665. 10.1152/ajprenal.1999.276.5.F658 10330047

[B55] Smith D. E. Pavlova A. Berger U. V. Hediger M. A. Yang T. Huang Y. G. (1998). Tubular Localization and Tissue Distribution of Peptide Transporters in Rat Kidney. Pharm. Res. 15 (8), 1244–1249. 10.1023/a:1011996009332 9706056

[B56] Sorensen M. V. Grossmann S. Roesinger M. Gresko N. Todkar A. P. Barmettler G. (2013). Rapid Dephosphorylation of the Renal Sodium Chloride Cotransporter in Response to Oral Potassium Intake in Mice. Kidney Int. 83 (5), 811–824. 10.1038/ki.2013.14 23447069

[B57] Su Y.-F. Shyu Y.-C. Shen C.-K. J. Hwang J. (2012). Phosphorylation-Dependent SUMOylation of the Transcription Factor NF-E2. PLoS One 7 (9), e44608. 10.1371/journal.pone.0044608 22970264 PMC3438180

[B58] Szklarczyk D. Franceschini A. Wyder S. Forslund K. Heller D. Huerta-Cepas J. (2015). STRING V10: Protein-Protein Interaction Networks, Integrated over the Tree of Life. Nucleic Acids Res. 43 (Database issue), D447–D452. 10.1093/nar/gku1003 25352553 PMC4383874

[B59] Tammsalu T. Matic I. Jaffray E. G. Ibrahim A. F. M. Tatham M. H. Hay R. T. (2015). Proteome-Wide Identification of SUMO Modification Sites by Mass Spectrometry. Nat. Protoc. 10 (9), 1374–1388. 10.1038/nprot.2015.095 26292070

[B60] Tempé D. Piechaczyk M. Bossis G. (2008). SUMO under Stress. Biochem. Soc. Trans. 36 (Pt 5), 874–878. 10.1042/BST0360874 18793154

[B61] Tomasi M. L. Ramani K. (2018). SUMOylation and Phosphorylation Cross-Talk in Hepatocellular Carcinoma. Transl. Gastroenterol. Hepatol. 3, 20. 10.21037/tgh.2018.04.04 29780898 PMC5945704

[B62] Uzoma I. Hu J. Cox E. Xia S. Zhou J. Rho H.-S. (2018). Global Identification of Small Ubiquitin-Related Modifier (SUMO) Substrates Reveals Crosstalk between SUMOylation and Phosphorylation Promotes Cell Migration. Mol. Cell Proteom. 17 (5), 871–888. 10.1074/mcp.RA117.000014 PMC5930406 29438996

[B63] Wang L. Qian J. Yang Y. Gu C. (2021a). Novel Insights into the Impact of the SUMOylation Pathway in Hematological Malignancies (Review). Int. J. Oncol. 59 (3), 73. 10.3892/ijo.2021.5253 34368858 PMC8360622

[B64] Wang T. Wu J. Dong W. Wang M. Zhong X. Zhang W. (2021b). The MEK Inhibitor U0126 Ameliorates Diabetic Cardiomyopathy by Restricting XBP1’s Phosphorylation Dependent SUMOylation. Int. J. Biol. Sci. 17 (12), 2984–2999. 10.7150/ijbs.60459 34421344 PMC8375222

[B65] Wang Y. Yu J. (2021). Dissecting Multiple Roles of SUMOylation in Prostate Cancer. Cancer Lett. 521, 88–97. 10.1016/j.canlet.2021.08.034 34464672

[B66] Wu Q. Aroankins T. S. Cheng L. Fenton R. A. (2019). SUMOylation Landscape of Renal Cortical Collecting Duct Cells. J. Proteome Res. 18 (10), 3640–3648. 10.1021/acs.jproteome.9b00306 31502464

[B67] Wu Q. Moeller H. B. Stevens D. A. Sanchez-Hodge R. Childers G. Kortenoeven M. L. A. (2018). CHIP Regulates Aquaporin-2 Quality Control and Body Water Homeostasis. J. Am. Soc. Nephrol. 29 (3), 936–948. 10.1681/ASN.2017050526 29242247 PMC5827596

[B68] Yang X.-J. Chiang C.-M. (2013). Sumoylation in Gene Regulation, Human Disease, and Therapeutic Action. F1000Prime Rep. 5, 45. 10.12703/P5-45 24273646 PMC3816760

[B69] Yokota K. Shibata H. Kurihara I. Kobayashi S. Suda N. Murai-Takeda A. (2007). Coactivation of the N-Terminal Transactivation of Mineralocorticoid Receptor by Ubc9. J. Biol. Chem. 282 (3), 1998–2010. 10.1074/jbc.M607741200 17105732

[B70] Zhou W. Ryan J. J. Zhou H. (2004). Global Analyses of Sumoylated Proteins in *Saccharomyces cerevisiae* . J. Biol. Chem. 279 (31), 32262–32268. 10.1074/jbc.M404173200 15166219 PMC2810850

[B71] Zhu B. Zhu L. Xia L. Xiong Y. Yin Q. Rui K. (2020). Roles of Ubiquitination and Deubiquitination in Regulating Dendritic Cell Maturation and Function. Front. Immunol. 11 (2957), 586613. 10.3389/fimmu.2020.586613 33329564 PMC7717991

