# Supplementary material for: The Hydrogen-Coupled Oligopeptide Membrane Cotransporter Pept2 is SUMOylated in Kidney Distal Convoluted Tubule Cells
Source: Front Mol Biosci. 2021 Nov 22;8:790606. doi: 10.3389/fmolb.2021.790606 (PMC8646034; doi:10.3389/fmolb.2021.790606)

Supplemental Information for

The hydrogen-coupled oligopeptide membrane cotransporter Pept2 is SUMOylated in kidney distal convoluted tubule cells

Takwa S. Aroankins[1, 2], Sathish K. Murali[1], Robert A. Fenton[1], Qi Wu[1]*

1) *Department of Biomedicine, Aarhus University, Aarhus DK-8000, Denmark*
2) *Department of Anesthesiology and Intensive Care, Sahlgrenska University Hospital, Sahlgrenska Academy at University of Gothenburg, Gothenburg, Sweden*

Wild-type flag-tagged mouse Pept2 DNA sequence………………………………..…page 2

Mutant K139R flag-tagged mouse Pept2 DNA sequence…………………….…..page 3

Figure S1.…………………………………………………...…………...….………..page 4

Figure S2……………………………………………………………………...…….. page 5

Figure S3…………………………………………………………………….... page 6

Annexes……………………………………………………………………...page 7-13

Wild-type flag-tagged mouse Pept2 DNA sequence:

```
ATGAACCCGTTTCAGAAAAACGAAAGCAAAGAAACCCTGTTTAGCCCGGTGAGCACC
GAAGAAATGCTGCCGGGCCCGCCGAGCCCGCCGAAAAAAAGCACCCCGAAACTGTTT
GGCAGCAGCTATCCGCTGAGCATTGCGTTTATTGTGGTGAACGAATTTTGCGAACGCTT
TAGCTATTATGGCATGAAAGCGGTGCTGACCCTGTATTTTCTGTATTTTCTGCATTGGA
ACGAAGATACCAGCACCAGCGTGTATCATGCGTTTAGCAGCCTGTGCTATTTTACCCCG
ATTCTGGGCGCGGCGATTGCGGATAGCTGGCTGGGCAAATTTAAAACCATTATTTATCT
GAGCCTGGTGTATGTGCTGGGCCATGTGTTTAAAAGCCTGGGCGCGATTCCGATTCTG
GGCGGCAAAATGCTGCATACCATTCTGAGCCTGGTGGGCCTGAGCCTGATTGCGCTGG
GCACCGGCGGCATTAAACCGTGCGTGGCGGCGTTTGGCGGCGATCAGTTTGAAGAAGA
ACATGCGGAAGCGCGCACCCGCTATTTTAGCGTGTTTTATCTGAGCATTAACGCGGGC
AGCCTGATTAGCACCTTTATTACCCCGATGCTGCGCGGCGATGTGAAATGCTTTGGCGA
AGATTGCTATGCGCTGGCGTTTGGCATTCCGGGCCTGCTGATGGTGCTGGCGCTGGTGG
TGTTTGCGATGGGCAGCAAAATGTATCGCAAACCGCCGCCGGAAGGCAACATTGTGGC
GCAGGTGACCAAATGCATTTGGTTTGCGATTTGCAACCGCTTTCGCAACCGCAGCGAA
GATATTCCGAAACGCCAGCATTGGCTGGATTGGGCGGCGGAAAAATATCCGAAACATC
TGATTATGGATGTGAAAGCGCTGACCCGCATTCTGTTTCTGTATATTCCGCTGCCGATG
TTTTGGGCGCTGCTGGATCAGCAGGGCAGCCGCTGGACCCTGCAGGCGAACAAAATGG
ATGGCGATCTGGGCTTTTTTGTGCTGCAGCCGGATCAGATGCAGGTGCTGAACCCGTTT
CTGGTGCTGGTGTTTATTCCGCTGTTTGATCTGGTGATTTATCGCCTGATTAGCAAATG
CGGCGTGAACTTTAGCAGCCTGCGCAAAATGGCGGTGGGCATGATTCTGGCGTGCCTG
GCGTTTGCGGTGGCGGCGCTGGTGGAAATTAAAATTAACGGCATGATTCATCCGCAGC
CGGCGAGCCAGGAAATTTTTCTGCAGGTGCTGAACCTGGCGGATGGCGAAATTGAAGT
GACCGTGCAGGGCAACCGCAACAACCCGCTGCTGGTGGAAAGCATTAGCAGCTTTCAG
AACACCACCCATTATAGCAAACTGCGCCTGGAAACCAAAAGCCAGGATCTGCATTTTC
ATCTGAAATATAACAACCTGAGCGTGCATAACGAATATAGCGTGGAAGAAAAAAACT
GCTATCAGCTGGTGGTGCATGAAAACGGCGAAAGCCTGAGCAGCATGCTGGTGAAAG
ATACCGGCATTAAACCGGCGAACGGCATGACCGCGATTCGCTTTATTAACACCCTGCA
TAAAGATATGAACATTAGCCTGGATGCGAACGCGCCGCTGAGCGTGGGCAAAGATTAT
GGCGTGAGCGAATATCGCACCGTGCAGCGCGGCAAATATCCGGCGGTGCATTGCGAA
ACCGAAGATAACGTGTTTAGCCTGAACCTGGGCCAGCTGGATTTTGGCACCACCTATC
TGTTTGTGATTACCAACATTACCAACCGCGGCCTGCAGGCGTGGAAAGCGGAAGATAT
TCCGGCGAACAAACTGAGCATTGCGTGGCAGCTGCCGCAGTATGTGCTGGTGACCGCG
GCGGAAGTGATGTTTAGCGTGACCGGCCTGGAATTTAGCTATAGCCAGGCGCCGAGCA
GCATGAAAAGCGTGCTGCAGGCGGCGTGGCTGCTGACCGTGGCGGTGGGCAACATTAT
TGTGCTGATTGTGGCGCAGTTTAGCGGCCTGGTGCAGTGGGCGGAATTTGTGCTGTTTA
GCTGCCTGCTGCTGGTGGTGTGCCTGATTTTTAGCGTGATGGGCTATTATTATGTGCCG
CTGAAAAGCGAAGGCATTCATGAAGCGACCGAAAAACAGATTCCGCATATTCAGGGC
AACATGATTAACCTGGAAACCAAAAACACCCGCCTGTGA
```

Mutant K139R flag-tagged mouse Pept2 DNA sequence:

```
ATGAACCCGTTTCAGAAAAACGAAAGCAAAGAAACCCTGTTTAGCCCGGTGAGCACC
GAAGAAATGCTGCCGGGCCCGCCGAGCCCGCCGAAAAAAAGCACCCCGAAACTGTTT
GGCAGCAGCTATCCGCTGAGCATTGCGTTTATTGTGGTGAACGAATTTTGCGAACGCTT
TAGCTATTATGGCATGAAAGCGGTGCTGACCCTGTATTTTCTGTATTTTCTGCATTGGA
ACGAAGATACCAGCACCAGCGTGTATCATGCGTTTAGCAGCCTGTGCTATTTTACCCCG
ATTCTGGGCGCGGCGATTGCGGATAGCTGGCTGGGCAAATTTAAAACCATTATTTATCT
GAGCCTGGTGTATGTGCTGGGCCATGTGTTTAAAAGCCTGGGCGCGATTCCGATTCTG
GGCGGCAGAATGCTGCATACCATTCTGAGCCTGGTGGGCCTGAGCCTGATTGCGCTGG
GCACCGGCGGCATTAAACCGTGCGTGGCGGCGTTTGGCGGCGATCAGTTTGAAGAAGA
ACATGCGGAAGCGCGCACCCGCTATTTTAGCGTGTTTTATCTGAGCATTAACGCGGGC
AGCCTGATTAGCACCTTTATTACCCCGATGCTGCGCGGCGATGTGAAATGCTTTGGCGA
AGATTGCTATGCGCTGGCGTTTGGCATTCCGGGCCTGCTGATGGTGCTGGCGCTGGTGG
TGTTTGCGATGGGCAGCAAAATGTATCGCAAACCGCCGCCGGAAGGCAACATTGTGGC
GCAGGTGACCAAATGCATTTGGTTTGCGATTTGCAACCGCTTTCGCAACCGCAGCGAA
GATATTCCGAAACGCCAGCATTGGCTGGATTGGGCGGCGGAAAAATATCCGAAACATC
TGATTATGGATGTGAAAGCGCTGACCCGCATTCTGTTTCTGTATATTCCGCTGCCGATG
TTTTGGGCGCTGCTGGATCAGCAGGGCAGCCGCTGGACCCTGCAGGCGAACAAAATGG
ATGGCGATCTGGGCTTTTTTGTGCTGCAGCCGGATCAGATGCAGGTGCTGAACCCGTTT
CTGGTGCTGGTGTTTATTCCGCTGTTTGATCTGGTGATTTATCGCCTGATTAGCAAATG
CGGCGTGAACTTTAGCAGCCTGCGCAAAATGGCGGTGGGCATGATTCTGGCGTGCCTG
GCGTTTGCGGTGGCGGCGCTGGTGGAAATTAAAATTAACGGCATGATTCATCCGCAGC
CGGCGAGCCAGGAAATTTTTCTGCAGGTGCTGAACCTGGCGGATGGCGAAATTGAAGT
GACCGTGCAGGGCAACCGCAACAACCCGCTGCTGGTGGAAAGCATTAGCAGCTTTCAG
AACACCACCCATTATAGCAAACTGCGCCTGGAAACCAAAAGCCAGGATCTGCATTTTC
ATCTGAAATATAACAACCTGAGCGTGCATAACGAATATAGCGTGGAAGAAAAAAACT
GCTATCAGCTGGTGGTGCATGAAAACGGCGAAAGCCTGAGCAGCATGCTGGTGAAAG
ATACCGGCATTAAACCGGCGAACGGCATGACCGCGATTCGCTTTATTAACACCCTGCA
TAAAGATATGAACATTAGCCTGGATGCGAACGCGCCGCTGAGCGTGGGCAAAGATTAT
GGCGTGAGCGAATATCGCACCGTGCAGCGCGGCAAATATCCGGCGGTGCATTGCGAA
ACCGAAGATAACGTGTTTAGCCTGAACCTGGGCCAGCTGGATTTTGGCACCACCTATC
TGTTTGTGATTACCAACATTACCAACCGCGGCCTGCAGGCGTGGAAAGCGGAAGATAT
TCCGGCGAACAAACTGAGCATTGCGTGGCAGCTGCCGCAGTATGTGCTGGTGACCGCG
GCGGAAGTGATGTTTAGCGTGACCGGCCTGGAATTTAGCTATAGCCAGGCGCCGAGCA
GCATGAAAAGCGTGCTGCAGGCGGCGTGGCTGCTGACCGTGGCGGTGGGCAACATTAT
TGTGCTGATTGTGGCGCAGTTTAGCGGCCTGGTGCAGTGGGCGGAATTTGTGCTGTTTA
GCTGCCTGCTGCTGGTGGTGTGCCTGATTTTTAGCGTGATGGGCTATTATTATGTGCCG
CTGAAAAGCGAAGGCATTCATGAAGCGACCGAAAAACAGATTCCGCATATTCAGGGC
AACATGATTAACCTGGAAACCAAAAACACCCGCCTGTGA
```

Transporters	Ncor2 *¥	Ncor1*¥	Arntl*¥	Smad4φ§	Nfat5*§	Pax2*¥	Ets1φ§	Tcf7l2φ§	Ehf*¥	Yy1φ§	Elf3φ¥	Gata3φ¥	Rreb1*§	Sp1φ¥	Score
Slc2a4	0.92	0.92													0.92
Glra1			0.91												0.905
Scn4a				0.86											0.858
Scn5a				0.84											0.844
Slc14a2					0.84										0.84
Pkd1						0.84									0.836
Slc6a12					0.82										0.816
Abcb1a							0.81								0.812
Slc30a8								0.8							0.802
Aqp2					0.78							0.71			0.78
Cldn7									0.76		0.71				0.755
Slc26a3										0.74					0.739
Trpc6					0.74										0.739
Slc6a6					0.74										0.738
Htr1a												0.71			0.714
Slc39a1													0.71		0.709
Abca1														0.702	0.702
Slco4c1												0.7			0.701
Slc31a1														0.7	0.7
Score	0.92	0.915	0.905	0.858	0.84	0.836	0.812	0.802	0.755	0.739	0.714	0.714	0.709	0.702	0.92

Transcription factors

* SUMO1 unique
‡ SUMO2 unique
φ SUMOylated by both SUMO1 and SUMO2

¥ Single SUMOylation
§ Multiple SUMOylation

Figure S1. Interaction analysis between SUMOylated transcription factors found in this study and known membrane transporters and channels by STRING. Only interactions with a high confidence (interaction score ≥0.7) were retained.

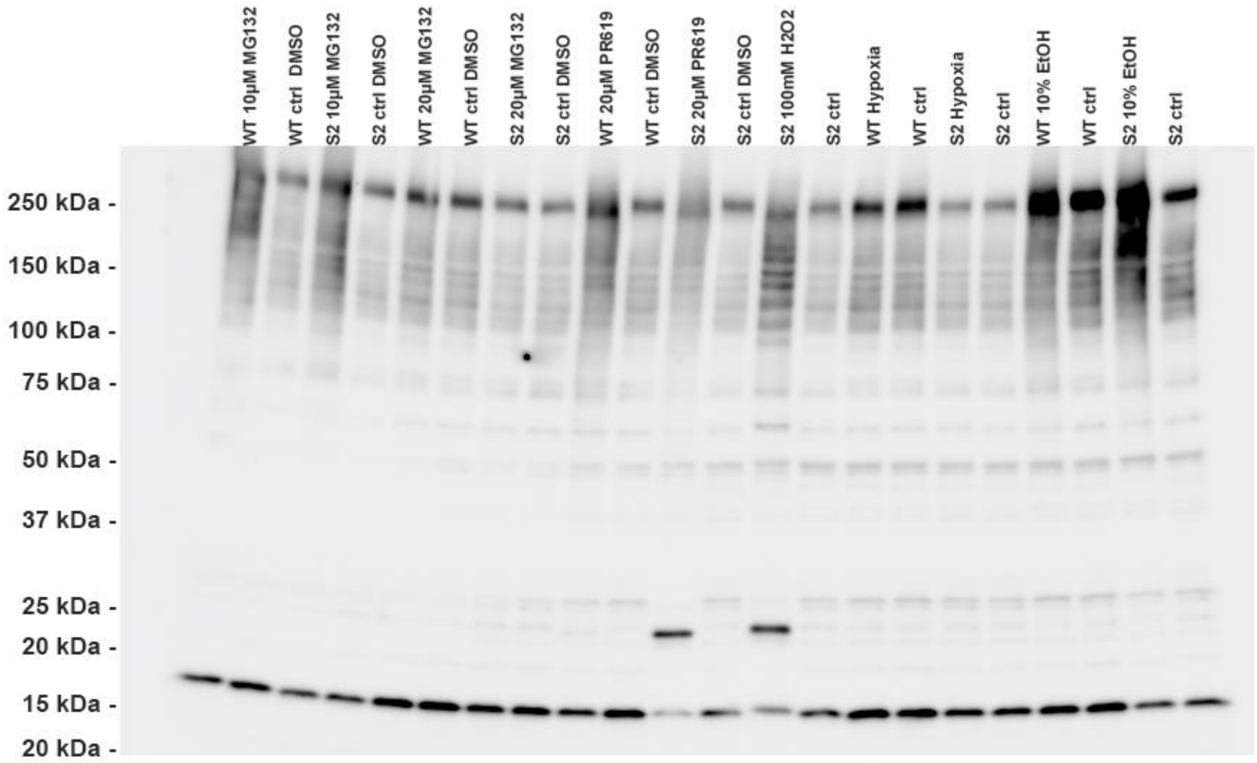

Figure S2. Characterization of SUMOylation in mpkDCT cells. Wild-type (WT) and 6xHis-SUMO2^{T90K} mutated mpkDCT cells (mpkDCT-SUMO2^{T90K}) were treated with various chemicals or pathophysiological conditions known to increase protein SUMOylation. After inhibition of deSUMOylation enzymes with PR619 or MG132, or inducing cell stress with hydrogen peroxide, SUMOylation was increased. Neither 10% ethanol or hypoxia treatment showed any major ability to increase SUMOylated proteins.

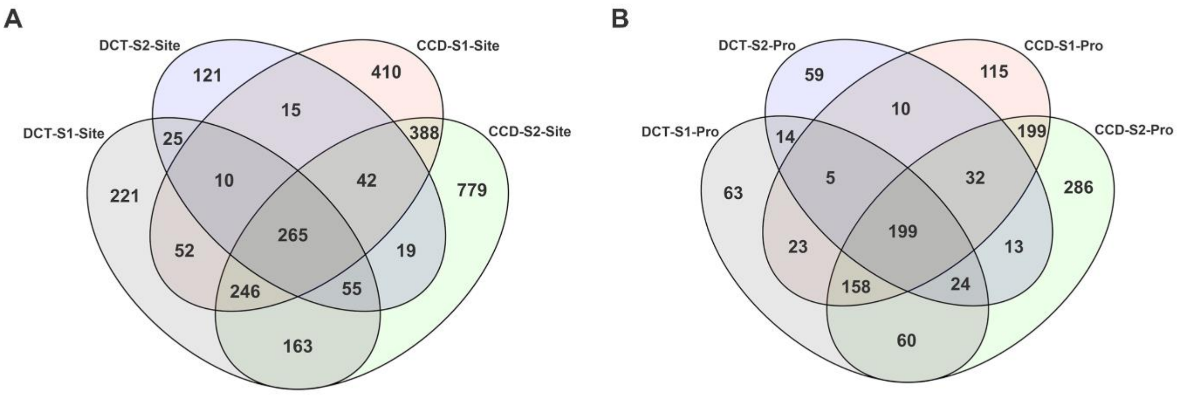

Figure S3. Venn diagram to compare A) SUMO1 and SUMO2 sites and B) SUMO1 and SUMO2 proteins between this study and our previous kidney cortical collecting duct cell (CCD) database (Wu *et al*., (2019). SUMOylation Landscape of Renal Cortical Collecting Duct Cells. *J Proteome Res* 18(10), 3640-3648).

ANNEX: All included western blots in this file consist of the entire membrane that was incubated with the antibody/developed with ECL. Areas used in specific figures are highlighted.

Figure 1A – SUMO1

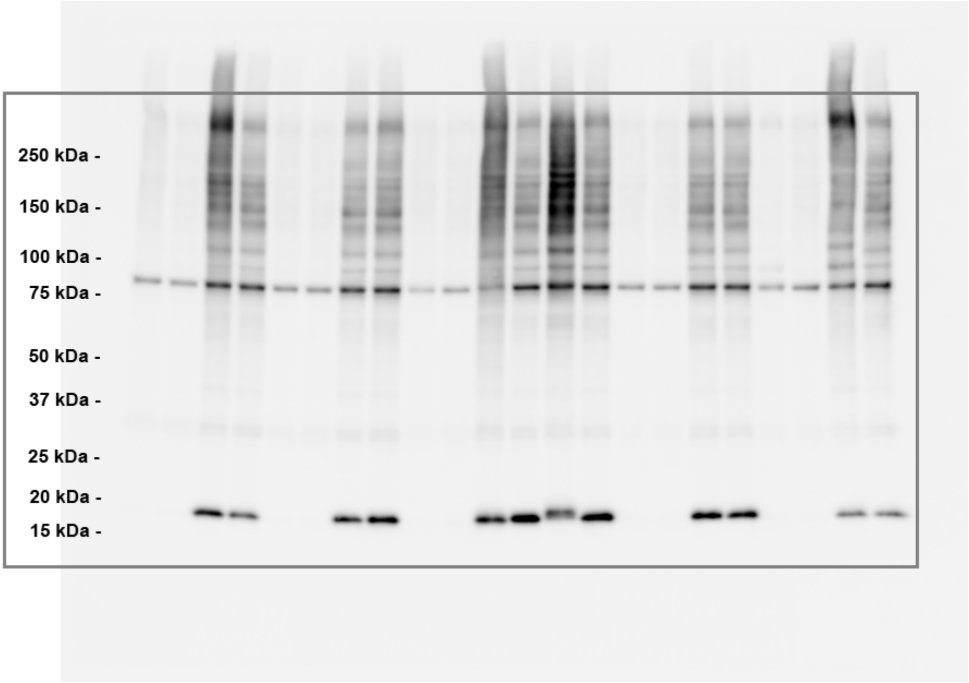

Figure 1A – Actin

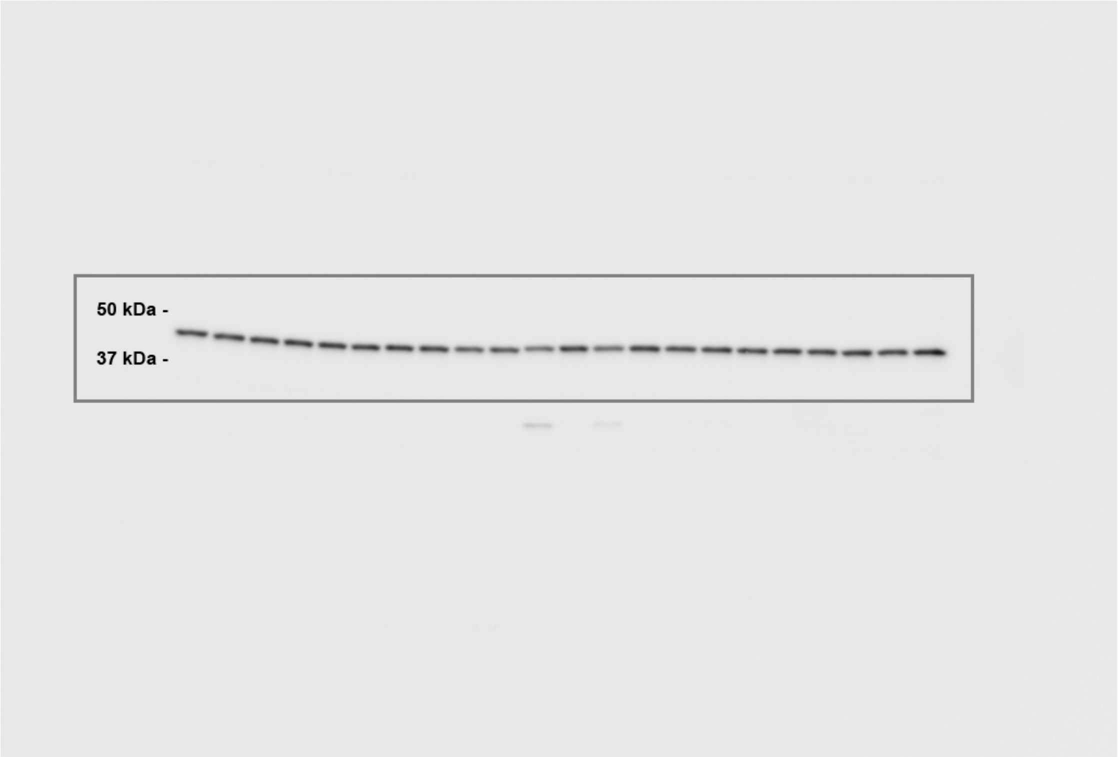

Figure 1B – SUMO1

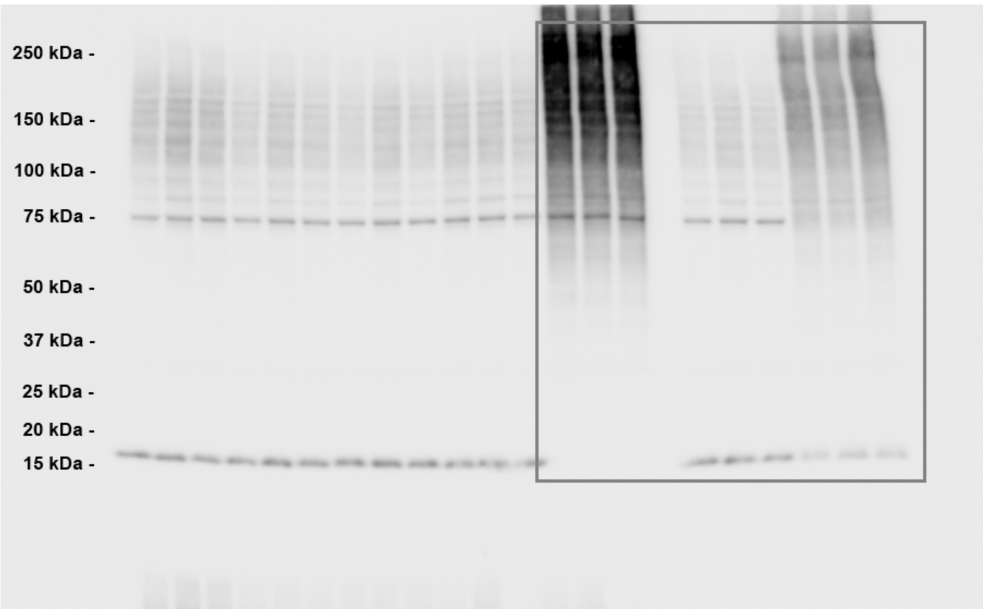

Figure 5A – Pept2

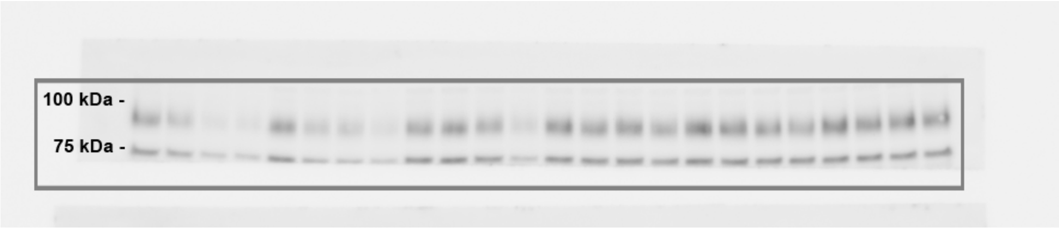

Figure 5A – αENaC

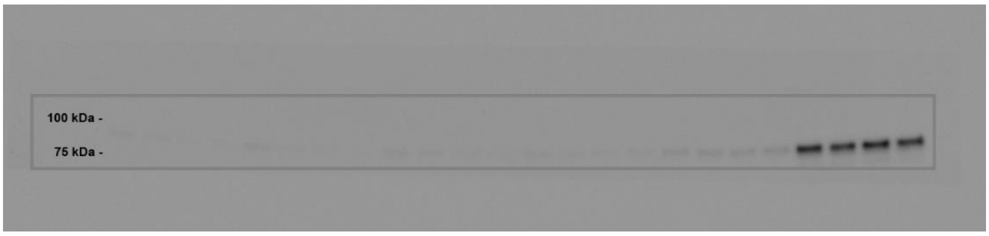

Figure 5A – Actin

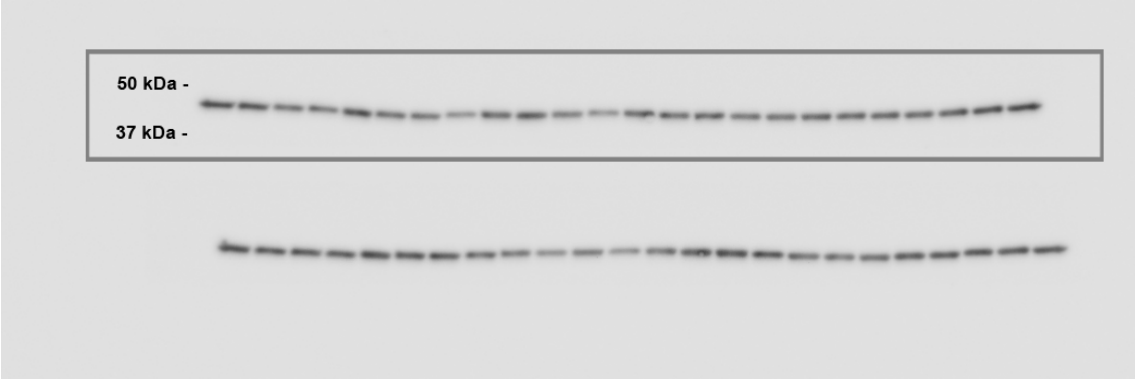

Figure 5B – Pept2

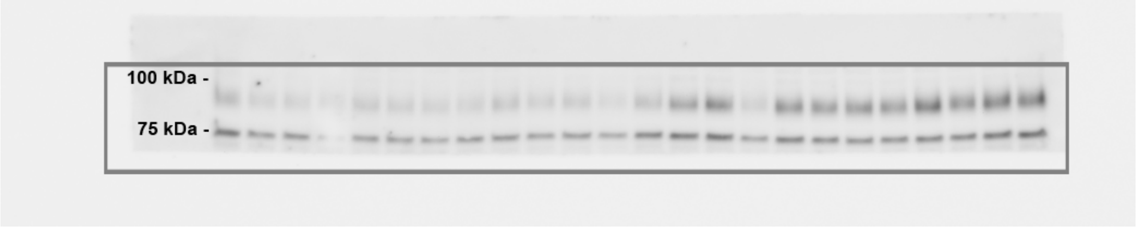

Figure 5B – αENaC

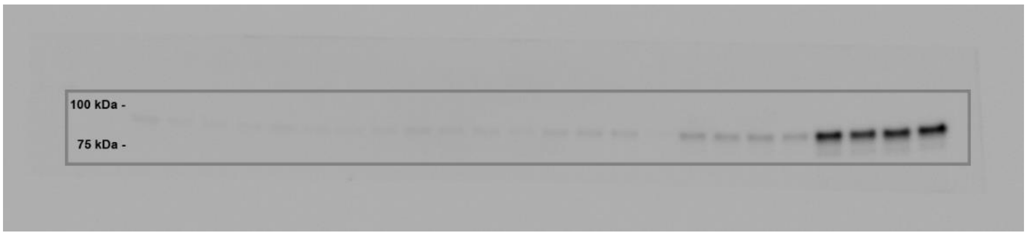

Figure 5B – Actin

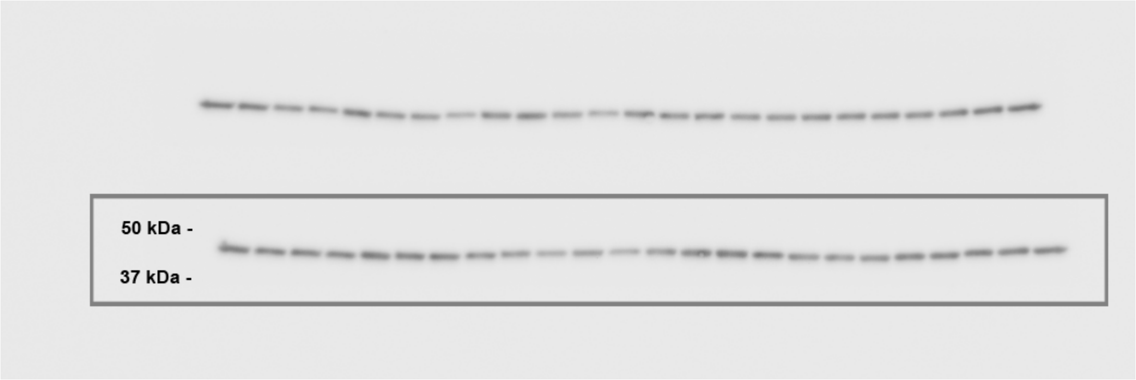

Figure 5C – Pept2

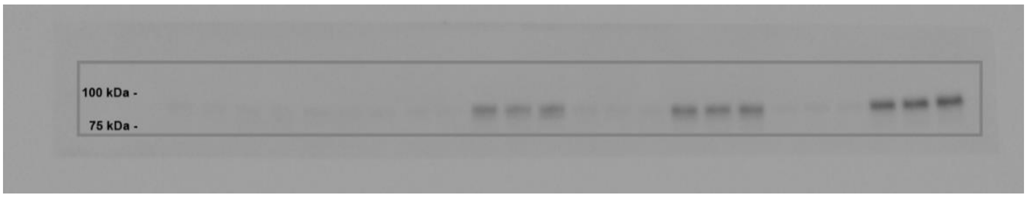

Figure 5C – αENaC

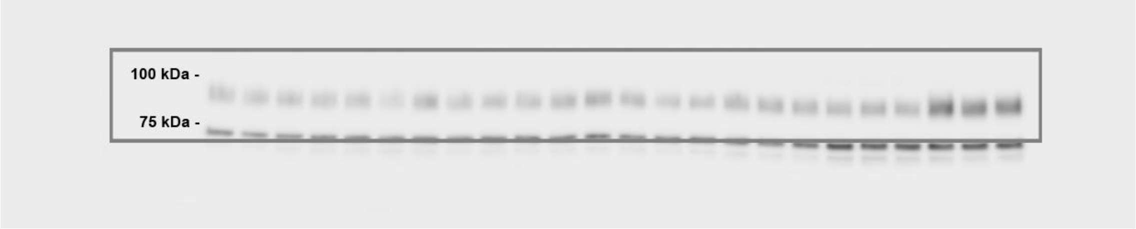

Figure 5C – Actin

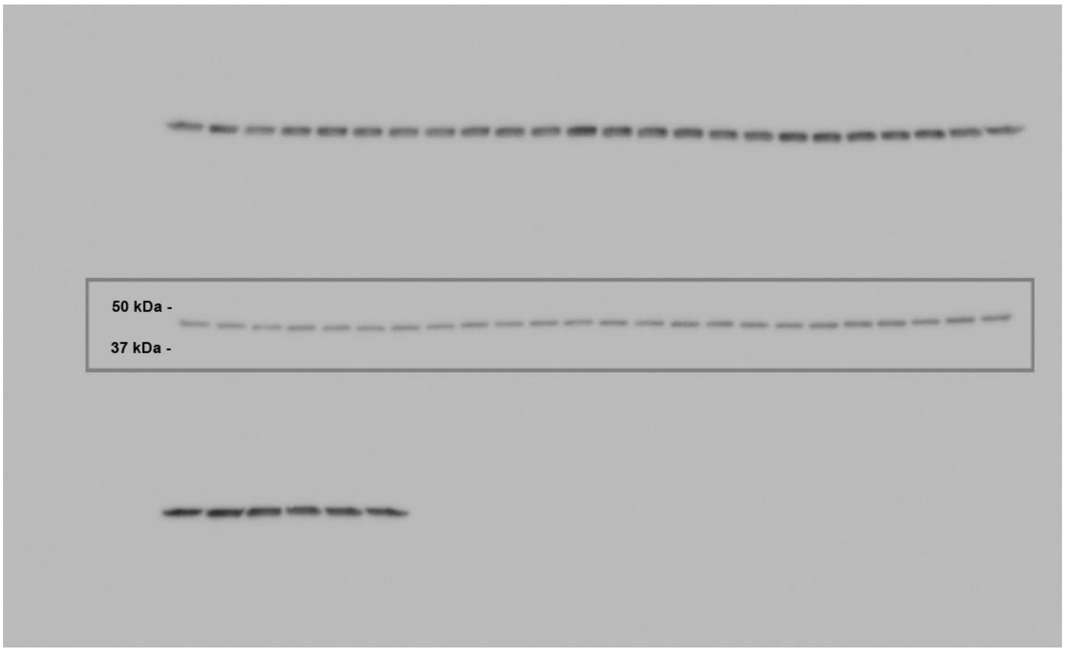

Figure 6 – Biotinylated Pept2

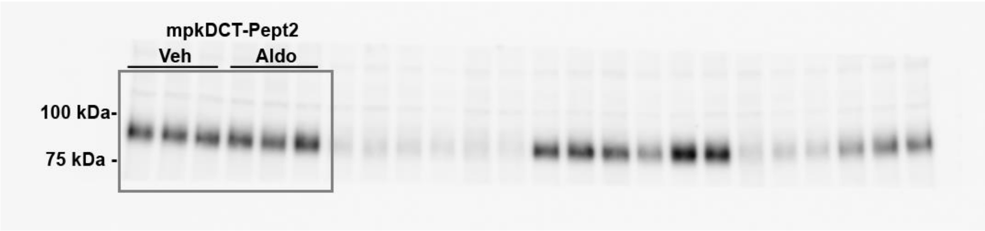

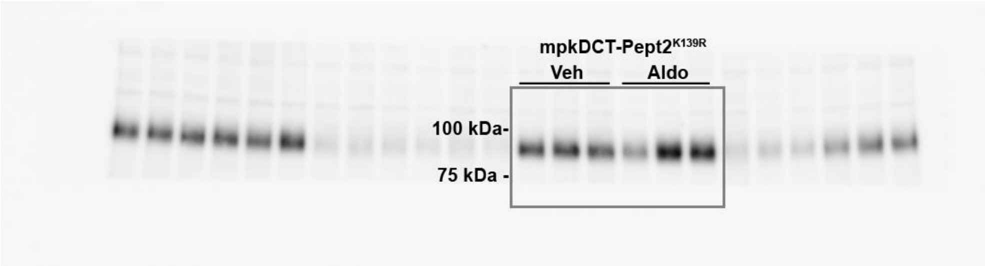

Figure 6 – Total Pept2

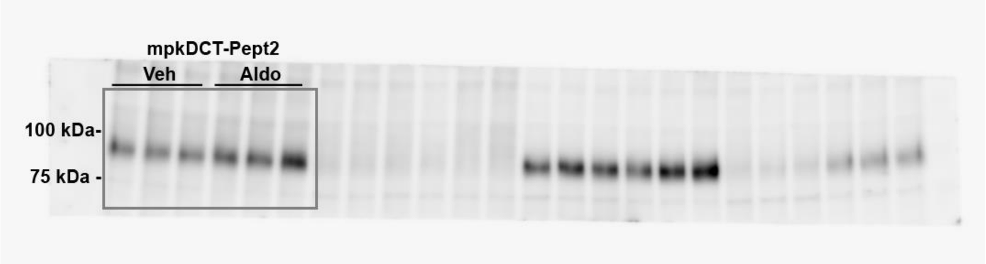

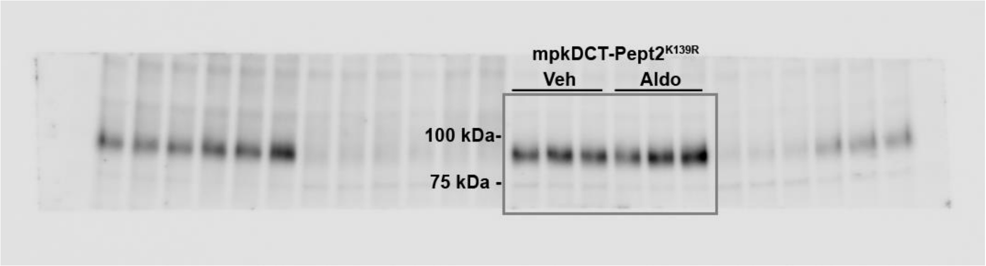

Figure 6 – Total Actin

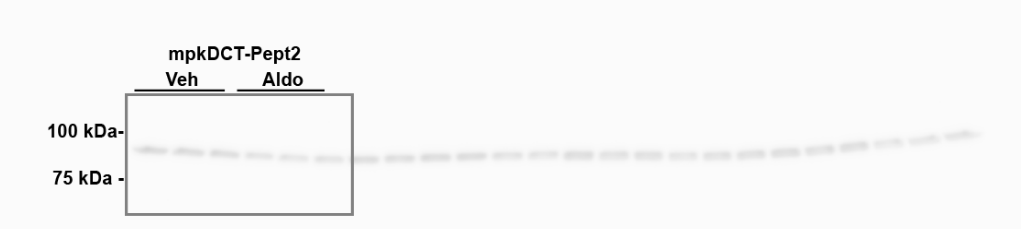

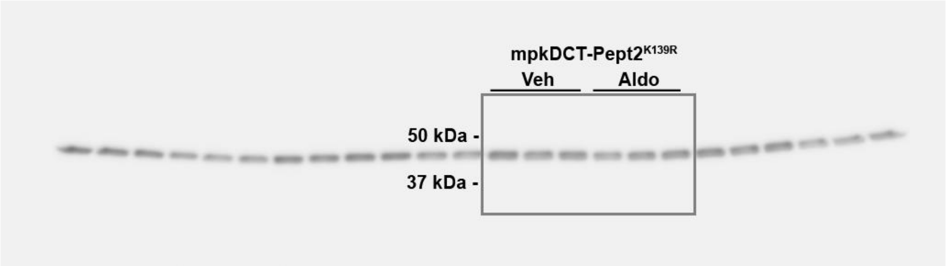

Figure 6 – Proteasome 20s on mpkDCT-Pept2 Apical surface biotinylation experiment

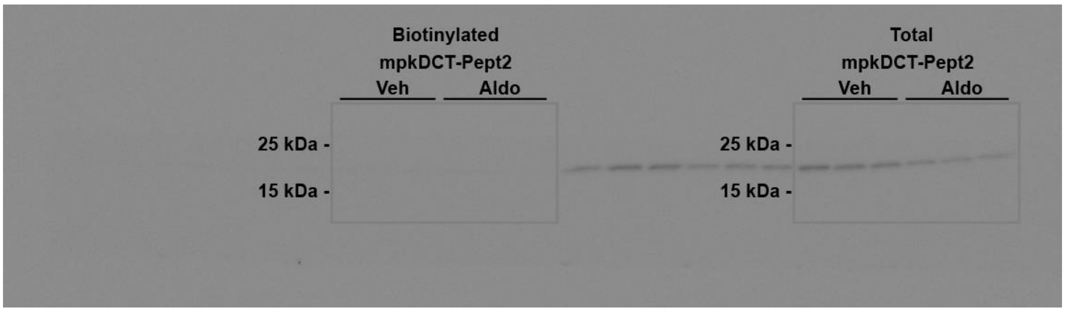

Figure 6 – Data analysis from four experiments

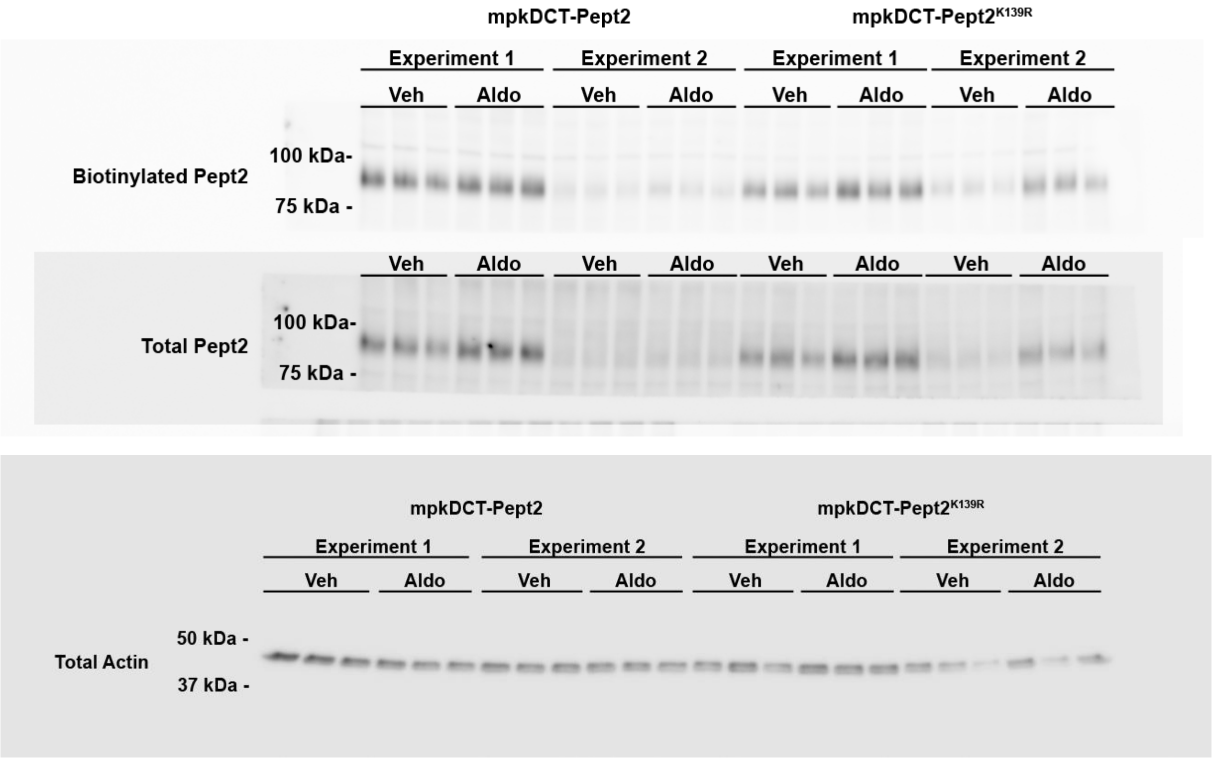

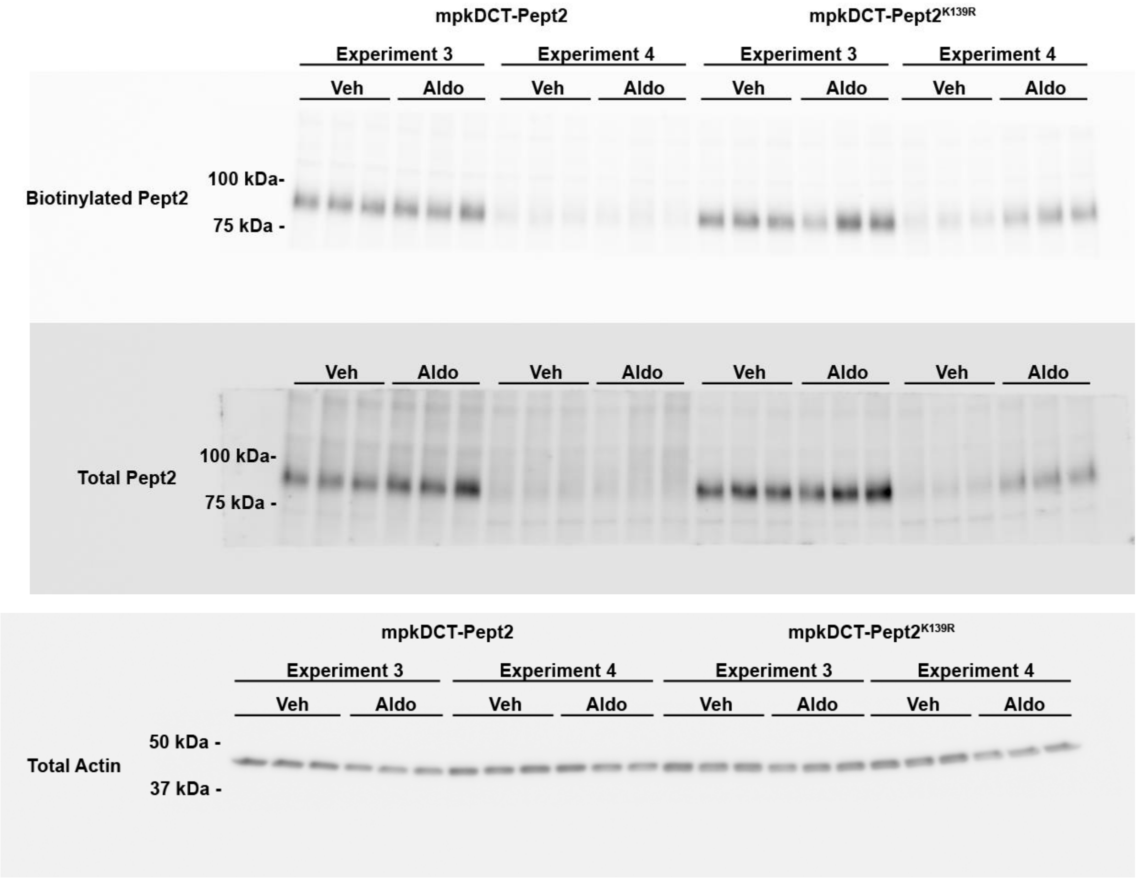

Figure S1 – SUMO2 stress experiment

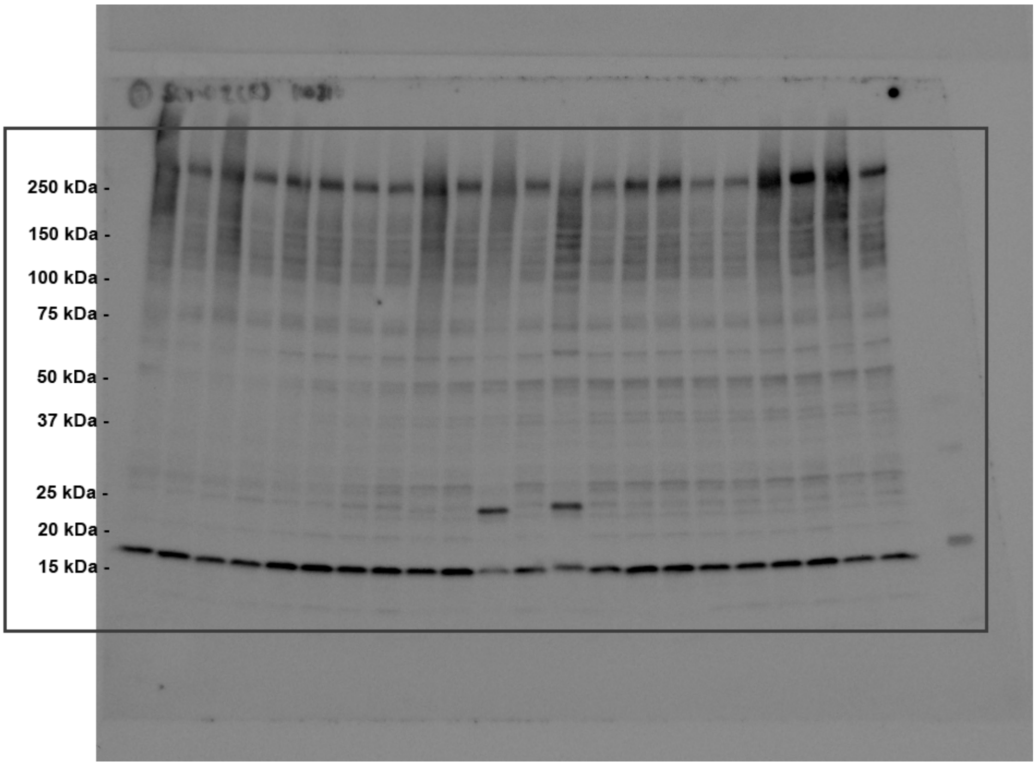

Supplement: Supplementary file 1 [file DataSheet1.PDF]
